# Plants Metabolites: Possibility of Natural Therapeutics Against the COVID-19 Pandemic

**DOI:** 10.3389/fmed.2020.00444

**Published:** 2020-08-07

**Authors:** Farhana Rumzum Bhuiyan, Sabbir Howlader, Topu Raihan, Mahmudul Hasan

**Affiliations:** ^1^Department of Botany, University of Chittagong, Chittagong, Bangladesh; ^2^Laboratory of Biotechnology and Molecular Biology, Department of Botany, University of Chittagong, Chittagong, Bangladesh; ^3^Department of Applied Chemistry and Chemical Engineering, University of Chittagong, Chittagong, Bangladesh; ^4^Department of Genetic Engineering and Biotechnology, Shahjalal University of Science and Technology, Sylhet, Bangladesh; ^5^Department of Pharmaceuticals and Industrial Biotechnology, Sylhet Agricultural University, Sylhet, Bangladesh

**Keywords:** medicinal plants, secondary metabolites, antiviral activities, natural therapeutics/alternative medicine, drug discovery, COVID-19

## Abstract

COVID-19, a disease induced by SARS-CoV-2 (Severe Acute Respiratory Syndrome Coronavirus-2), has been the cause of a worldwide pandemic. Though extensive research works have been reported in recent days on the development of effective therapeutics against this global health crisis, there is still no approved therapy against SARS-CoV-2. In the present study, plant-synthesized secondary metabolites (PSMs) have been prioritized to make a review focusing on the efficacy of plant-originated therapeutics for the treatment of COVID-19. Plant metabolites are a source of countless medicinal compounds, while the diversity of multidimensional chemical structures has made them superior to treat serious diseases. Some have already been reported as promising alternative medicines and lead compounds for drug repurposing and discovery. The versatility of secondary metabolites may provide novel antibiotics to tackle MDR (Multi-Drug Resistant) microbes too. This review attempted to find out plant metabolites that have the therapeutic potential to treat a wide range of viral pathogens. The study includes the search of remedies belonging to plant families, susceptible viral candidates, antiviral assays, and the mode of therapeutic action; this attempt resulted in the collection of an enormous number of natural therapeutics that might be suggested for the treatment of COVID-19. About 219 plants from 83 families were found to have antiviral activity. Among them, 149 plants from 71 families were screened for the identification of the major plant secondary metabolites (PSMs) that might be effective for this pandemic. Our investigation revealed that the proposed plant metabolites can serve as potential anti- SARS-CoV-2 lead molecules for further optimization and drug development processes to combat COVID-19 and future pandemics caused by viruses. This review will stimulate further analysis by the scientific community and boost antiviral plant-based research followed by novel drug designing.

## Introduction

Coronaviruses comprise a group of large, enveloped, positive-sensed, single-stranded RNA viruses that damage the respiratory tract of mammals including humans, bats, and other animals, leading to infections in the respiratory tract (1–5). The Coronavirus disease 2019 (COVID-19), initially called 2019 novel coronavirus (2019-nCoV), is an agile respiratory disease caused by a novel coronavirus primarily detected in Wuhan, China (6, 7). Now, it has spread to 216 countries and caused the death of more than 0.5 million people worldwide and was declared as a pandemic by the World Health Organization (WHO) (8, 9). Seven types of human coronaviruses have been reported so far, including HCoV-OC43, HCoV-229E, HCoV-HKU1, HCoV-NL63, severe acute respiratory syndrome (SARS)-CoV, Middle East respiratory syndrome (MERS-CoV), and 2019-novel coronavirus nCoV (10). Among them, MERS-CoV, SARS-CoV, and nCoV have taken the concern of scientists worldwide. In 2003, the severe acute respiratory syndrome (SARS) outbreak occurred in Guangdong (southern China) (6, 11) which infected 8,000 people and resulted in 800 deaths in 26 countries. Only a decade later, another coronavirus has attacked the world and caused another devastating outbreak, MERS, which infected 2,494 people and caused the deaths of 858 worldwide (12, 13). However, the COVID-19 pandemic caused by SARS CoV-2 resulted in remarkable levels of morbidity and mortality all over the world. Initially China, followed by the USA, Italy, France, Iran, Spain, Russia, Turkey, and the UK became hotspots for SARS CoV-2. The virus hotspot has now moved to Latin America and, at this time, Brazil, Mexico, and Peru are the new hotspots of SARS CoV-2. The important aspects of the pathobiology, a viral response phase, and a hyperbolic host response phase are linked with the morbidity and mortality in COVID-19 patients (14). However, the increased cytokine levels (IL-6, IL-10, and TNF-α), lymphopenia (in CD4+ and CD8+ T cells), and decreased IFN-γ expression in CD4+ T cells are the more risky and possibly life-threatening events related to severe COVID-19 (15–17). The infection rate of COVID-19 is increasing gradually but scientists have not been able to suggest any specific drug, vaccine, or any other certified therapeutic agents against SARS-CoV-2, which consequently leads to the significant morbidity and mortality.

On the other hand, plants have been essential to human welfare for their uses as therapeutics since ancient times (18, 19). According to the WHO, about 80% of the world's population depends on medicinal plants or herbs to fulfill their medicinal needs (20–22). A significant amount of antiviral compounds produced from numerous kinds of plants have been used in many studies (23–25). Researchers all around the world are screening therapeutic drugs from existing antiviral plant secondary metabolites (PSMs) and are also trying to find novel compounds from medicinal plants [(26–159); Supplementary Table 1] to avert this global crisis. Plant metabolites can halt the activity of enzymes involved in the replication cycle of CoVs including papain-like protease and 3CL protease, halt the fusion of the S protein of coronaviruses and ACE2 of the host, and also inhibit cellular signaling pathways (123, 144, 160). Screening from existing PSMs, researchers have been trying to find novel compounds from medicinal plants to prevent numerous diseases, including COVID-19 (Supplementary Table 1). Therefore, the current manuscript aims to describe potential metabolites from plant sources that have antiviral properties that might be aligned for the alternative approach against COVID-19. Hence, understanding the structure, life cycle, pathogenicity, cell signaling, epidemiology of the recently emerging virus, drug targets, and drug discovery process have become very important issues to find specific/effective therapeutics.

## Epidemiology, Genomic Organization, and Life Cycle of SARS CoV-2

In December 2019, SARS CoV-2, one of the most devastating viral outbreaks since SARS CoV and MERS, originated from Wuhan city seafood market in China (161–163). The virus was found to be transmitted through close contact with infected people or through exposure to coughing, sneezing, and respiratory droplets (164, 165). It has already been reported to have spread to 216 countries and caused more than 0.5 million deaths. Brazil is now the new hotspot for SARS CoV-2 after the USA, Russia, France, Italy, Germany, Spain, and the UK, where more than 11 million people are infected (166, 167).

**Figure 1:**
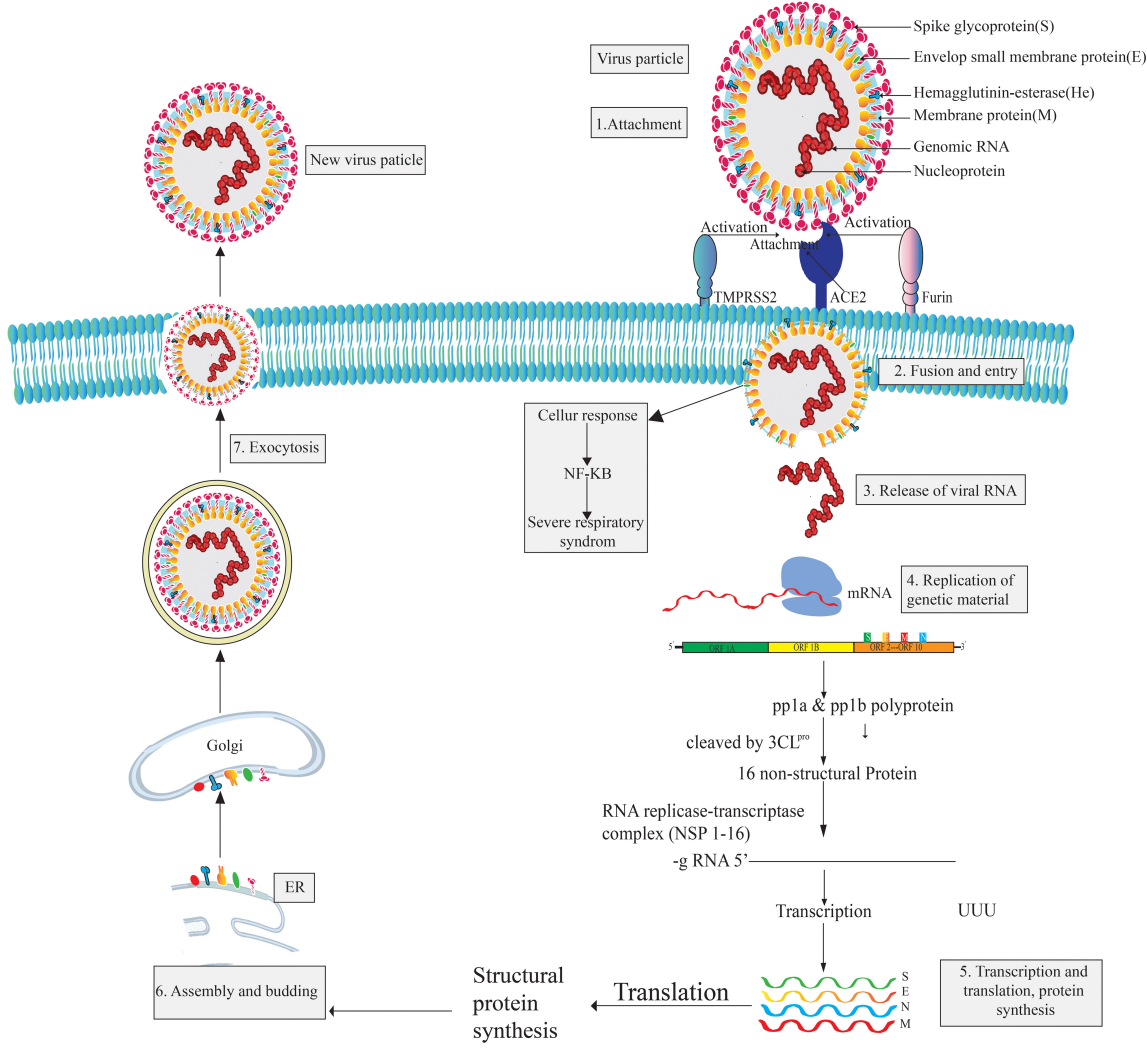
Structure, genomic organization, life cycle, and drug targets of SARS CoV-2.

The pleomorphic or spherical shaped SARS COV-2 has a single-stranded RNA genome of 26.4–31.7 kb in length and a crown-like glycoproteins on its surface (168–173). It is more similar to SARS CoV (over 80%) than MERS (174, 175). However, the RNA genome of CoV-2 is considered as one of the largest genomes compared to those of other RNA viruses (176, 177). The largest open reading frame, ORF1ab, encodes non-structural proteins while the remaining ORFs encode four structural proteins, namely the envelope glycoprotein or spike protein (S), envelope (E) protein, membrane (M) protein, and nucleocapsid (N) protein. The S protein mediates attachment to the host cell while the E protein is involved in virus assembly, membrane permeability of the host cell, and virus-host cell interaction. The M protein is known as a central organizer for the coronavirus assembly and the nucleocapsid (N) protein is usually involved in the processing of helical ribonucleocapsid complex, including some accessory proteins (172, 178). Six types of mutations are found in the genome of SARS CoV-2 while three mutations have been reported in *orf 1ab* gene, two mutation in *S* gene, and the final one in the *orf 7b* and *orf 8* (174, 175). Proteomic analysis revealed that SARS CoV-2 is vastly homologous to SARS CoV but two proteins, *orf 8* and *orf 10*, are not homologous to SARS CoV (175). To complete its life cycle, SARS CoV-2 passes into the human body through the nose, mouth, or eyes and then attaches itself to the receptor-binding domain (RBD) using the surface glycoprotein (Spike-protein) of the virion which tries to attach with the hACE2 receptor (179, 180). The entry mechanism of SARS CoV-2 depends on cellular transmembrane serine protease 2 (TMPRSS2) and furin, along with viral receptor ACE2 (180–182). However, after the fusion of the SARS CoV-2 virion particle with the host cell membrane, the envelope and capsid part of the virus are removed. The virus releases its genetic material (RNA) into the host cell cytoplasm and acts as mRNA for the translation from ORF1a and ORF1ab to produce pp1a and pp1ab polypeptides (169, 183). Subsequently, chymotrypsin-like protease (3CL^pro^) slices these polypeptides into 16 non-structural proteins (NSPs) that are responsible for replication and transcription (184). Then, infected cells produce proteins when they become hijacked by SARS CoV-2. In this situation, the immune system supports the assembly of SARS CoV-2 into new copies of virion particles (185, 186). Freshly synthesized viral nucleic acids and proteins then assemble into the lumen of the ERGIC (Endoplasmic Reticulum Golgi Intermediate Compartment) and leave the cells through exocytosis [(187, 188); Figure 1]. Infected cells release virions and infect other human cells.

SARS-CoV-2 viral infection can be divided into three stages: the asymptomatic period, non-severe symptomatic period, and the severe infection stage (17, 189). SARS CoV-2 patients are reported to have a significant amount of cytokines and chemokines; the levels of cytokines are especially highly increased in patients admitted to ICUs (Intensive Care Unit) (190, 191). These significantly high levels are what results in a patient reaching a critical stage. However, the main mediator of SARS CoV-2, the spike glycoprotein, is found in two conformations (192) and the enzyme 3CL^pro^ of SARS-CoV-2 share a 99.02% sequence identity with 3CL^pro^ of SARS-CoV, which is also highly similar to bat SARS CoV 3CL^pro^ (193). SARS CoV-2 binds to the host cell receptor with a higher affinity than SARS CoV (194). SARS CoV-2 has shown some strategic alteration with the substrate-binding site of bat SARS CoV-2 and 12 point-mutations are found in SARS CoV-2 compared to SARS CoV. Mutations disrupt the significant hydrogen bonds and modify the receptor binding site (RBS) of SARS-CoV-2 3CL^pro^. However, the occurrence of recurrent mutations can lead to new strains with alterations in virulence, which one of the reasons discovering a suitable vaccine to combat SARS CoV-2 is challenging (175, 195).

## Major Drug Targets of SARS CoV-2

A fundamental therapeutic approach to treat multi-viral infections is the interruption of human host-virus interactions (17). The major structural proteins of SARS CoV-2 can be obvious targets for drugs designed against COVID-19. In addition, 16 non-structural proteins (NSPs) can also be considered (169). However, the manifestation of recurrent recombination events is a major hindrance to develop SARS CoV-2 specific vaccines/drugs (176). Up-to-date studies revealed that, though SARS-CoV-2 and SARS-CoV identify a similar receptor (ACE2) in humans (194, 196), there is a noteworthy variation in the antigenicity between SARS-CoV and SARS-CoV-2 which has significance on the development of therapeutic options against this rapidly emerging virus (197). The SARS-CoV-2 spike protein exhibits a higher affinity to the ACE2 receptor in comparison to SARS-CoV, but hACE2 showed a lower binding affinity to RBD (Receptor Binding Domain) of SARS COV-2 when compared to SARS CoV (194, 198). The two most paramount enzymes of SARS CoV-2, proprotein convertase furin- potentiates cell fusion and serine protease TMPRSS2, are responsible for S-protein activation and are propitious drug targets for the treatment of COVID (180, 194, 199).

## Sars-CoV-2 and Searching for Effective Therapeutics

Though extensive research works are being continued for the development of effective vaccines or drug compounds against SARS-CoV-2, efficacious therapeutics have not yet been attained (200). Moreover, interferon therapies, monoclonal antibodies, oligonucleotide-based therapies, peptides, small-molecule drugs, and vaccines, are regarded as some strategic approaches for controlling or preventing COVID-19 (201, 202). Existing drugs can be used as the first-line treatment for coronavirus outbreaks, but this is not the ultimate solution to eradicate the disease (203). Therefore, the development of therapeutic drugs for the treatment of the COVID-19 outbreak have gathered considerable attention. Scientists from different fields are trying to figure out the way to develop therapeutics. However, experimental implications of drug recombination might be both expensive and time-consuming, whereas computational evaluation may bring about testable hypotheses for systematic drug recombination (174).

## PSMs Can be Effective Over Synthetic Drugs Against SARS CoV-2

Though there are approved, repurposed drugs currently in clinical use, there is still an urgent need for specific antiviral therapeutics and vaccines (199). Bioengineered and vectored antibodies and therapies based on cytokines and nucleic acid which target virus gene expression have been found as promising to treat coronavirus infections (204). For example, the repurposing drugs, including favipiravir, remdesivir, lopinavir, ritonavir, nebulized α-interferon, chloroquine, hydroxychloroquine, ribavirin, and interferon (IFN), have been shown to be effective for the treatment of COVID-19. Apart from this, some therapeutics are in clinical trials, such as peptide vaccine (mRNA-1273) (198) and antibody therapies (205). Recently, plasma therapy showed promising results for COVID-19 treatment (206, 207). But, application of these synthetic drugs are not efficient as they exhibit adverse direct or indirect side effects [(208–220); Table 1]. In addition, scientists all around the world are trying to find out some prominent drug and multi-epitope vaccine candidates against this deadly virus using various kinds of immuno-informatics approaches (221, 222). Therefore, the urgent need for safe, effective, and inexpensive therapies/drugs with negligible side effects against COVID-19 is imperative.

**Table 1:** Recently used synthetic drugs and their side effects.

**Drug**	**Side effects**	**References**
Arbidol	Side effects in children include sensitization to the drug	(208)
Darunavir	Liver problems and severe skin reactions or rash	(209)
Flavipir	–	(210)
Hydroxychloroquine	One of the most serious side effects of hydroxychloroquine is a risk of heart rhythm problems, which can result in heart failure and in some cases death. Hydroxychloroquine can upset the stomach. Severe, permanent damage to the retina has been reported with the use of hydroxychloroquine	(211)
Ivermectin	Eye or eyelid irritation, pain, redness, or swelling	(212)
Lopinavir	Drowsiness, dizziness, a bad taste in the mouth, and trouble sleeping	(213)
Loprazolam	Paradoxical increase in aggression, lightheadedness, blood disorders, and jaundice	(214)
Lurasidone	Drowsiness, lightheadedness, weight gain, mask-like facial expression, and agitation	(214)
Oseltamivir	Phlegm-producing cough, wheezing, abdominal or stomach cramps or tenderness, bloating	(215)
Remdesivir	Increased liver enzyme levels that may indicate possible liver damage	(209, 211)
Ribavirin	Allergic reaction, anemia, stabbing chest pain, wheezing	(220)
Ritonavir	Diarrhea, nausea, vomiting, heartburn, stomach pain, dizziness, tiredness	(215)
Salmeterol	Hoarseness, throat irritation, rapid heartbeat, cough, dry mouth/throat, or upset stomach	(217)
Saquinavir	Hyperglycemia, increased bleeding in people with hemophilia, increases in the levels of certain fats	(209)
Talampicillin	–	(214)
Teicoplanin	Maculopapular or erythematous rash and drug-related fever	(218)
Andrographolide (PSM)	–	(219)
Rubitecan	–	(214)

PSMs are a source of natural antiviral compounds that could be an effective option, as most of them are safer and more cost-effective compared to orthodox drugs (223), though some PSMs are toxic too. The dependency on and popularity of plant-based drugs are increasing day by day (224). Due to the presence of multiple compounds in crude plant extracts, it can be either beneficial or not, depending on the amounts used each time; if properly regulated, better activity might be shown. It was also found that crude extracts can target multiple sites at a time in a virion particle (225). However, this is yet to be tested against SARS-CoV-2. PSMs can affect the disruption of cell membrane functions and structures (226), interference with intermediary metabolisms (227), interruption of DNA/RNA synthesis and function (228), interruption of normal cell communication (quorum sensing) (229), and the induction of coagulation of cytoplasmic constituents (230). Different kinds of plant metabolites act against SARS CoV (Supplementary Table 1). Plant-based products affect several key events in the pathogenic process. For example, curcumin is effective for its antineoplastic, anti-proliferative, anti-aging, anti-inflammatory, anti-angiogenic, antiviral and anti-oxidant effects, and can regulate redox status, protein kinases, transcription factors, adhesion molecules, and cytokines in the human body (231). *In silico* analysis revealed that anti-SARS CoV PSMs could be one of the most valuable drug targets against SARS CoV-2 [(232); Table 2]. A huge amount of plant metabolites have remained unexplored due to the extensive process of isolation of the target compound. Now, various types of modern techniques have been developed for the isolation of lead compounds from crude extracts including maceration, percolation, decoction, reflux extraction, soxhlet extraction, pressurized liquid extraction, supercritical fluid extraction, ultrasound assisted extraction, microwave-assisted extraction, pulsed electric field extraction, enzyme assisted extraction, hydro distillation, and steam distillation (179). These techniques can lead us to find out novel anti-SARS CoV-2 compounds earlier than traditional techniques. In addition, plant metabolomics are used as a tool for the discovery of novel drugs from plant resources (262, 263).

**Table 2:** Probable drug candidates against SARS CoV-2 obtained through virtual screening.

**Drug targets**	**Major metabolites**	**References**
**ANTIVIRAL PSMs THAT CAN INHIBIT SARS CoV-2 AT DIFFERENT TARGET**		
Spike protein	Magnoflorine, tinosponone, cirsimaritin, chrysoeriol, vasicinone, quercetin, luteolin	(233)
Spike protein	Epigallocatechingallate (EGCG), curcumin, apigenin, chrysophanol	(234)
Spike protein, main protease	Spike protein, main protease	(235)
Spike protein and ACE-2	Hesperidin, emodin, and chrysin	(236)
Spike protein and ACE-2	Curcumin, nimbin, withaferin A, piperine, mangiferin, thebaine, berberine, and andrographolide	(222)
Spike protein and ACE-2	Chebulagic acid	(237)
Spike protein, MPro, and RdRp	Silybin, withaferin A, cordioside, catechin, and quercetin	(238)
RdRp	Protopine, allocryptopine, and (±) 6-acetonyldihydrochelerythrine	(239)
Main Protease (MPro)	Crocin, digitoxigenin, and b–eudesmol	(240)
Main Protease (MPro)	Oolonghomobisflavan-A, theasinensin D, theaflavin-30-O-gallate	(241)
Main Protease (MPro)	Andrographolide	(219)
Main Protease (MPro)	Hispidin, lepidine E, and folic acid	(242)
Main Protease (MPro)	Ursolic acid, carvacrol, and oleanolic acid	(243)
Main Protease (MPro)	Hypericin, cyanidin 3-glucoside, baicalin, glabridin	(244)
Main Protease (MPro)	Cetylglucopetunidin, isoxanthohumol, and ellagic acid	(245)
Main Protease (MPro)	Benzylidenechromanones	(246)
Main Protease (MPro)	Carnosol, arjunglucoside-I, and rosmanol	(247)
Main Protease (MPro)	Leucoefdin	(248)
Main Protease (MPro)	(1E,6E)-1,2,6,7-tetrahydroxy-1,7-bis(4-hydroxy-3-methoxyphenyl)hepta-1,6-diene-3,5-dione) and (4Z,6E)-1,5-dihydroxy-1,7-bis(4-hydroxyphenyl)hepta-4,6-dien-3-one	(249)
Mpro and ACE2	Quercetin 3-glucuronide-7-glucoside, and Quercetin 3-vicianoside	(250)
Mpro, hACE-2 and RdRp	d-Viniferin, myricitrin, chrysanthemin, myritilin, taiwanhomoflavone A, lactucopicrin 15-oxalate, nympholide A, afzelin, biorobin, hesperidin, and phyllaemblicin B	(251)
Mpro, spike protein, and non-structural proteins (NSP-9, 15)	Arzanol, ferulic acid, genistein, resveratrol, rosmanol	(252)
ACE-2 receptor	Resveratrol, pterostilbene, pinosylvin, piceatannol	(253)
ACE-2 receptor	Isothymol, chloroquine, captopril	(254)
ACE-2 receptor	Resveratrol, quercetin, luteolin, naringenin, zingiberene, and gallic acid	(222)
Envelope protein	Belachinal, macaflavanone E, vibsanol B	(249)
PLpro, 3CLpro	Cryptotanshinone, quercetin, tanshinone IIa, coumaroyltyramine, N-cis-feruloyltyramine	(178)
PLpro, 3CLpro, RdRp, and spike protein	Andrographolide (AGP1), 14-deoxy 11,12-didehydro andrographolide (AGP2), neoandrographolide (AGP3), and 14-deoxy andrographolide (AGP4)	(255)
3CLpro	10-hydroxyusambarensine, cryptoquindoline, 6-oxoisoiguesterin, 22-hydroxyhopan-3-one, cryptospirolepine, isoiguesterin, and 20-epibryonolic acid	(256)
3CLpro	Flavone and coumarine	(209)
3CLpro	Myricitrin, methyl rosmarinat, calceolarioside B, licoleafol, amaranthin, colistin	(191)
6LU7 and 6Y2E proteases	Apigenin, glabridin, glycoumarin, oleanolic acid, glucobrassicin	(257)
Transmembrane protease serine 2 (TMPRSS2)	Withanone and withaferin-A	(258)
Membrane (M) and Envelope (E) proteins	Nimbolin A, nimocin, and cycloartanols	(259)
**ANTIVIRAL PSMs THAT CAN INHIBIT SARS CoV-2 AT DIFFERENT LIFE CYCLE**		
Viral attachment	Phytoestrogens (diadiazin, genistein, formontein, and biochanin A), chlorogenic acid, linolenic acid, palmitic acid, caffeic acid, caffeic acid phenethyl ester, hydroxytyrosol, cis-p-Coumaric acid, cinnamaldehyde, thymoquinone, and some physiological hormones such as estrogens, progesterone, testosterone, and cholesterol	(260)
Entry	Dihydrotanshinone – 1, desmethoxyreserpine	(241)
Multiplication	Betulinic acid, desmethoxyreserpine, lignan, sugiol	(241)
Viraus–host interaction	Dithymoquinone (DTQ)	(261)

## PSMs Having Antiviral Properties as Alternatives to Synthetic Drugs and Hope For CoVID-19

Plants produce diversified low molecular weight PSMs to protect them from different herbivores and microbes (264). Before the discovery of allopathic drugs, these leading natural sources were extensively used for treating several kinds of human diseases (265, 266). Due to the increased resistance of microbial pathogens against allopathic drugs, researchers have now returned to natural resources, focusing especially on plant metabolites, to find out lead compounds to fight against human pathogens (175). Moreover, about 35% of the global medicine market (which accounts for 1.1 trillion US dollars) have been shared by medicinal products prepared using natural plants or herbs (265). Investigations are undergoing for the finding of novel and modern drugs from numerous herbal preparations to fight against this microbial resistance war. Many similarities have been found between SARS CoV and SARS CoV-2 (both of them belong to beta family, containing the same genetic material-RNA, and using the same receptor for viral attachment-ACE2, with an 86% identity and 96% similarity of genome, with almost the same pathogenesis). Thus, previously reported antiviral plant metabolites for SARS CoV can be considered as emerging drug candidates for COVID-19. Right now, the setbacks arising from viral infection around the world have placed budget constraints on researchers trying to discover effective antiviral drugs. However, some PSMs have already shown anti-SARS CoV activity where other antiviral activities are also reported (Supplementary Table 1). These results suggest that there is a scope to find alternative medicines and specific compounds. So, plants could be a vital resource in the fight against COVID-19. Our study suggests that around 76 natural metabolites from different plant species can be efficiently active against COVID-19 (Table 3 and Supplementary Figure 1).

**Table 3:** Probable promising secondary metabolites of medicinal plants against COVID-19.

	**Compounds**	**Plant source**	**Family**	**References**
1.	Diterpneoid	*Andrographis paniculata*	Acanthaceae	(26)
2.	Alkaloids, flavonoids, and coumarins	*Sambucus nigra*	Adoxaceae	(29)
3.	Alkaloids, anthraquinones, glycosides, flavonoids, saponins, phenols, terpenoids, sugar bearing compound, protein, thiols, and inferences	*Iresine herbstii*	Amaranthaceae	(31)
4.	Tannins, Flavonoids, Terpenes, and Saponins	*Anacardium occidentale*	Anacardiaceae	(33)
5.	Tannins, gallic acid, flavonoids like quercetin and quercitrin, phenolics, triterpenes	*Rhus aromatica*	Anacardiaceae	(34)
6.	Gallic acid, quercetin, kaempferol, glycosides	*Rhus parviflora*	Anacardiaceae	(35)
7.	Tannins and flavonoids	*Spondias lutea*	Anacardiaceae	(33)
8.	Flavonoids	*Spondias lutea* L.	Anacardiaceae	(33)
9.	Apigenin and luteolin	*Arisaema tortuosum*	Araceae	(40)
10.	Phenolic acids, flavonoids (apigenin, apigeninglucoside, luteolin, cirsiliol, diosmetin), lignans, terpenic lactones, and alkamides	*Achillea fragrantissima*	Asteraceae	(47, 48)
11.	Flavonoids, clerodane diterpenoids, phenolics, hydroxycinnamic acids	*Baccharis gaudichaudiana DC*	Asteraceae	(49)
12.	Diterpenoids	*Baccharis spicata (Lam.) Baill*	Asteraceae	(49)
13.	Triterpenoids, Steroids	*Bidens subalternans DC*	Asteraceae	(49)
14.	Flavonoid glycosides and caffeoyl quinic acids	*Eupatorium perfoliatum*	Asteraceae	(50)
15.	Flavonoids and terpenes	*Jasonia montana*	Asteraceae	(47)
16.	Phenylpropanoids, flavonoids, essential oils, polyphenols, tannins, triterpenes	*Pluchea sagittalis (Lam.) Cabrera*	Asteraceae	(49)
17.	Silymarin, quercetin, and kaempferol	*Silybum marianum*	Asteraceae	(51)
18.	terpenoids, flavonoids, essential oils	*Tagetes minuta* L.	Asteraceae	(49)
19.	phenolic acids (chlorogenic acids), and sesquiterpene lactones (parthenolide)	*Tanacetum parthenium*	Asteraceae	(52)
20.	Flavonoids, D-glucopyranoside, quercetin, luteolin	*Taraxacum officinale*	Asteraceae	(53)
21.	Flavonoids (apigenin, quercetin, kaempferol, falcarinol, selinene, limonene, and zerumbone)	*Tridax procumbens*	Asteraceae	(55)
22.	Carbohydrates, lipids, proteins, alkaloids, flavonoids, saponins, and organic acids	*Balanites aegyptiaca*	Balanitaceae	(56, 57)
23.	Icariin and quercetin	*Epimedium koreanum Nakai*	Berberidaceae	(58)
24.	Flavonoids (quercetin, isoquercetin, and rutin)	*Capparis sinaica*	Capparaceae	(47, 64)
25.	Tannins, flavonoids, carbohydrates and/or glycosides, resins, sterol, saponins, and alkaloids	*Capparis sinaica*	Capparaceae	(47, 65)
26.	Natural lupane triterpenoids	*Cassine xylocarpa*	Celastraceae	(67)
27.	Pentacyclic lupane-type triterpenoids	*Maytenus cuzcoina*	Celastraceae	(67)
28.	Flavonoids, terpenoids, alkaloids, tannins, glycosides, and saponins	*Combretum adenogonium*	Combretaceae	(72)
29.	Triterpenes, flavonoids, ellagitannins	*Terminalia mollis*	Combretaceae	(56, 73)
30.	Lignans, diterpenes, flavonoids, proanthocyanidins, and sterols	*Taxodium distichum*	Cupressaceae	(75)
31.	Monoterpenoids, sesquiterpenoids, triterpenoids, sterols, alkaloids, flavonoids, and phenolic compounds	*Cyperus rotundus*	Cyperaceae	(76)
32.	Protocatecuic acid, caffeic acid, epicatechin, rutin, resveratrol, quercitin, kaempferol	*Ephedra alata*	Ephedraceae	(47, 77)
33.	Isoflavonoid, indoles, phytosterols, polysaccharides, sesquiterpenes, alkaloids, glucans, and tannins	*Equisetum giganteum*	Equisetaceae	(78)
34.	Triterpenes and steroids	*Euphorbia denticulata*	Euphorbiaceae	(79)
35.	Tannins, diterpenes	*Euphorbia hirta*	Euphorbiaceae	(80)
36.	Diterpenoids, jatrophane-type diterpenoids, and coumarino-type lignoids, lathyrane-type diterpenoids, multifidone, multifidanol, and multifidenol	*Jatropha multifida*	Euphorbiaceae	(82)
37.	Flavonoid and polyphenol	*Acacia arabica*	Fabaceae	(83)
38.	Luteolin and vitexin	*Aspalathus linearis*	Fabaceae	(85)
39.	Saponins and flavonoids	*Vachellia nilotica*	Fabaceae	(87)
40.	Catechin, kaempferol, quercetin, 3,4′,7-trihydroxyl-3′,5-dimethoxyflavone, rutin, isorhamnetin, epicatechin, afzelechin, epiafzelechin, mesquitol, ophioglonin, aromadendrin, and phenol	*Acacia catechu*	Fabaceae	(88)
41.	Flavonoids, phenolics, and tannins	*Quercus persica*	Fagaceae	(90)
42.	Phenolic, flavonoid, and flavonol compounds	*Quercus persica*	Fagaceae	(90)
43.	Gallic acid, protocatechuic acid, corilagin, geraniin, ellagic acid, kaempferitrin, kaempferol 7-O-rhamnoside, quercetin, kaempferol	*Geranium thunbergii*	Geraniaceae	(91)
44.	Flavonoids (orientin and vicenin)	*Ocimum sanctum*	Lamiaceae	(26, 99)
45.	Terpenoid and polyphenol	*Ocimum sanctum*	Lamiaceae	(83)
46.	Baicalin, flavonoids	*Scutellaria baicalensis*	Lamiaceae	(104)
47.	Opuntin B, triterpene saponin, seroids, and phenylethanoids	*Lindernia crustacea*	Linderniaceae	(107)
48.	Quercetin 3-O-methyl ether (3MQ) and strychnobiflavone (SBF)	*Strychnos pseudoquina*	Loganiaceae	(108)
49.	Alkaloids, flavonoids, tannins, volatile oils, and glycosides	*Cissampelos pareira Linn*	Menispermaceae	(113)
50.	Flavonoids, tannins, terpenes, saponins, and nitrogenous compounds	*Artocarpus integrifolia*	Moraceae	(33)
51.	Flavonoids, rutin, kaempferol 3-O-rutinoside, and kaempferol 3-O-robinobioside	*Ficus benjamina*	Moraceae	(114)
52.	N-arginine, luteolin, caffeic acid	*Ficus carica*	Moraceae	(115)
53.	Flavonoids, tannins, saponins, alkaloids, and steroids/triterpenoids	*Ficus religiosa*	Moraceae	(116)
54.	Tannins, flavonoid, saponin, glycoside	*Ficus sycomorus*	Moraceae	(56, 118)
55.	Alkaloids, tannins, phenolics, and saponins	*Moringa peregrina*	Moringaceae	(47)
56.	Flavonoids	*Myristica fragrans*	Myristicaceae	(33)
57.	Tannins and flavonoids	*Psidium guajava*	Myrtaceae	(33)
58.	Sesquiterpenes, monoterpenes, hydrocarbon, and phenolic compounds, eugenyl acetate, eugenol, and β-caryophyllene	*Syzygium aromaticum* L.	Myrtaceae	(119)
59.	Paeoniflorin, monoterpene glycosides, albiflorin, benzoylpaeoniflorin, gallic acid, ethyl gallate	*Paeonia delavayi*	Paeoniaceae	(121)
60.	Flavonoids, tomentin A, B, C, D, and E	*Paulownia tomentosa*	Paulowniaceae	(123)
61.	Highly oxygenated norbisabolane sesquiterpenoids, phyllanthacidoid acid, methyl ester	*Phyllanthus acidus*	Phyllanthaceae	(124)
62.	Alkaloids, flavonoids, lignans, phenols, and terpenes	*Phyllanthus amarus*	Phyllanthaceae	(125)
63.	Geraniin, rutin, gallic acid, caffeolquinic acid, corilagen, galloylglucopyronoside, digalloylglucopyronoside, and quercetin glucoside	*Phyllanthus amarus*	Phyllanthaceae	(126)
64.	Geraniin, rutin, gallic acid, caffeolquinic acid, corilagen, galloylglucopyronoside, digalloylglucopyronoside, and quercetin glucoside	*Phyllanthus niruri*	Phyllanthaceae	(126)
65.	Trigalloylglucopyronoside, quercetin rhamnoside, geraniin, rutin, gallic acid, caffeolquinic acid, corilagen, galloylglucopyronoside, digalloylglucopyronoside, and quercetin glucoside	*Phyllanthus urinaria*	Phyllanthaceae	(126)
66.	Quercetin rhamnoside, geraniin, rutin, gallic acid, caffeolquinic acid, corilagen, galloylglucopyronoside, digalloylglucopyronoside, and quercetin glucoside	*Phyllanthus watsonii*	Phyllanthaceae	(126)
67.	Plumbagin, allicin, carbohydrates, flavonoids, proteins, saponins, fats and oils, alkaloids, steroids, phenols, and tannins	*Plumbago indica*	Plumbaginaceae	(129)
68.	Flavonoids (catechin, hyperoside, quercitrin, quercetin, and rutin), tannins, and triterpenoids	*Agrimonia pilosa*	Rosaceae	(135)
69.	Hydroxycinnamic acids, eriodictyol, isorhamnetin, quercetin, kaempferol, isorhamnetin, epicatechin, catechin	*Prunus dulcis*	Rosaceae	(136)
70.	Saponins, flavonoids, and alkaloids	*Pavetta tomentosa*	Rubiaceae	(138)
71.	Saponins, flavonoids, and alkaloids	*Tarenna asiatica*	Rubiaceae	(138)
72.	Triterpenes, tannins, flavonoids, and carbohydrates	*Dimocarpus longan*	Sapindaceae	(140)
73.	Organic acids, terpenoids, and flavonoids	*Illicium verum Hook. f*.	Schisandraceae	(142)
74.	Nilocitin, ellagic acid, gallic acid, flavonoids	*Tamarix nilotica*	Tamaricaceae	(47, 143)
75.	Diterpenoids, biflavonoids (biflavone amentoflavone, apigenin, luteolin, and quercetin)	*Torreya nucifera*	Taxaceae	(144)
76.	Friedelolactones, 2β-hydroxy-3, 4-seco-friedelolactone-27-oic acid flavonoids, coumarins, terpenoids, sterols, polypeptides	*Viola diffusa*	Violaceae	(147)

## Plant-Based Antiviral Compounds: Group Basis Mechanism of Action and PSMs Structure

A wide variety of antiviral compounds were found from 219 medicinal plants (26–159) belonging to 83 plant families (Supplementary Table 1). First and foremost are polyphenols, which contain multiple phenolic rings, and are classified as phenols, flavonoids, lignans, hydroxycinnamic acid, stilbenes, and hydroxybenzoic acid (267). We found polyphenols in numerous plants (Table 4) which exerted antiviral activity (269–271) against a wide range of viruses including HIV-1, HIV-2, HSV-1, HSV-2, Influenza virus, Dengue virus, HBV, HCV, Infectious bronchitis virus (IBV), Murbarg virus, Ebola virus, Newcastle disease virus (NDV), Poliomyelitis-1 virus, Lentivirus, and Coronavirus. Polyphenols work against coronaviruses using diverse mechanisms including actuating or inhibiting cellular signaling pathways or halting papain-like protease (PL^pro^) and 3-chymotripsin-like protease (3CL^pro^) enzyme (269, 272). Some polyphenol compounds (30-(3-methylbut-2-enyl)-30, 4-hydroxyisolonchocarpin, broussochalcone A, 4,7-trihydroxyflavane, broussochalcone B, papyriflavonol A, kazinol A, kazinol B, kazinol F, kazinol J, and broussoflavan A) isolated from *Broussonetia papyrifera* showed promising activity against SARS CoV. Higher efficiency against PL_pro_ as observed by these compounds though activity against M_pro_ or 3CL_pro_ is not up to the mark. Specially, papyriflavonol A possesses impressive activity against SARS CoV (IC_50_ 3.7, l M) (272). *In silico* analysis revealed that polyphenols can inhibit SARS CoV-2 Mpro and RdRp effectively (273, 274). In our study, we have found another widely distributed, low molecular weight phenolic compound named as a flavonoid which showed strong antiviral activity against SARS CoV, Influenza virus, HBV, HSV, HCV, HIV, Dengue virus, Simian virus, Human rotavirus, Bovine viral diarrhea virus, Poliomyelitis-1 virus, Vesicular stomatitis virus (VSV), and Newcastle disease virus (NDV) (Table 4). Flavonoid type compounds, such as apigenin and quercetin, showed activity against SARS CoV virion particles through the inhibition of M_pro_ enzymes with an IC_50_ of 38.4 ± 2.4 μM and 23.8 μM, respectively (144, 150, 275). According to *in silico* analysis, flavonoid compounds can terminate the activity of M_pro_ of SARS CoV-2 (276, 277).

**Table 4:** Major group basis antiviral PSMs obtained from medicinal plants.

**Major compounds**	**Plant source**	**Family**	**Target pathogen**	**References**
Polyphenols	*Avicennia marina*	Acanthaceae	Human immunodeficiency virus (HIV) and herpes simplex virus (HSV)	(27)
	*Sambucus nigra*	Adoxaceae	Dengue virus serotype-2 (DENV-2)	(29)
	*Sambucus nigra*	Adoxaceae	Infectious bronchitis virus (IBV)—chicken coronavirus	(30)
	*Iresine Herbstii*	Amaranthaceae	Newcastle disease virus (NDV)	(31)
	*Anacardium occidentale*	Anacardiaceae	Simian (SA-11) virus	(33)
	*Artocarpus integrifolia*	Moraceae	(SA-11) and human (HCR3) rotaviruses	(33)
	*Myristica fragrans*	Myristicaceae	Human (HCR3) rotaviruses	(33)
	*Psidium guajava*	Myrtaceae	Simian (SA-11) virus	(33)
	*Spondias lutea*	Anacardiaceae	Human (HCR3) rotaviruses	(33)
	*Spondias lutea L*.	Anacardiaceae	Simian (SA-11) and human (HCR3) rotaviruses	(33)
	*Rhus aromatica*	Anacardiaceae	HSV-1 and HSV-2	(34)
	*Rhus aromatica*	Anacardiaceae	HSV-1 and HSV-2	(34)
	*Rhus parviflora*	Anacardiaceae	HIV-1	(35)
	*Schinus terebinthifolia*	Anacardiaceae	HSV-1	(36)
	*Arisaema Tortuosum*	Araceae	Acyclovir-resistant HSV-2 and HSV-1	(40)
	*Jasonia montana*	Asteraceae	Poliomyelitis-1 virus	(47)
	*Baccharis gaudichaudiana DC*	Asteraceae	Bovine viral diarrhea virus, HSV-1, Poliovirus type 2 (PV-2), and vesicular stomatitis virus (VSV)	(49)
	*Pluchea sagittalis (Lam.) Cabrera*	Asteraceae	Bovine viral diarrhea virus (BVDV) (HSV-1), poliovirus type 2 (PV-2), and vesicular stomatitis virus (VSV)	(49)
	*Tagetes minuta L*	Asteraceae	Bovine viral diarrhea virus, HSV-1, poliovirus type 2 (PV-2), and vesicular stomatitis virus	(49)
	*Eupatorium perfoliatum*	Asteraceae	Influenza A virus (IAV) H1N1	(50)
	*Silybum marianum*	Asteraceae	Chikungunya virus (CHIKV), hepatitis C virus (HCV)	(51)
	*Tanacetum parthenium*	Asteraceae	HSV-1	(52)
	*Taraxacum officinale*	Asteraceae	HCV	(53)
	*Senna angustifolia*	Fabaceae	Dengue virus serotype-2 (DENV-2)	(55)
	*Tridax procumbers*	Asteraceae	Dengue virus serotype-2 (DENV-2)	(55)
	*Vernonia cinerea*	Asteraceae	Dengue virus serotype-2 (DENV-2)	(55)
	*Epimedium koreanum Nakai*	Berberidaceae	Porcine epidermic diarrhea virus (PEDV)	(58)
	*Canarium album (Lour.)*	Burseraceae	Influenza A virus (IAV)	(62)
Polyphenols	*Cistus incanus*	Cistaceae	HIV (clinical HIV-1 and HIV-2) and Filoviruses, Ebola, and Marburg virus	(69)
	*Combretum adenogonium*	Combretaceae	HIV-1	(72)
	*Cornus canadensis*	Cornaceae	HSV-1	(74)
	*Taxodium distichum*	Cupressaceae	Influenza A and B viruses	(75)
	*Cyperus rotundus*	Cyperaceae	HSV-1, HBV	(76)
	*Equisetum giganteum*	Equisetaceae	HSV-2	(78)
	*Euphorbia hirta*	Euphorbiaceae	HIV-1, HIV-2, SIV mac 251	(80)
	*Euphorbia sikkimensis*	Euphorbiaceae	HIV-1	(81)
	*Acacia arabica*	Fabaceae	Influenza A virus H9N2	(83)
	*Aspalathus linearis*	Fabaceae	Rhesus rotavirus (RRV), simian rotavirus (SA-11) infection	(85)
	*Vachellia nilotica*	Fabaceae	HSV-2	(87)
	*Acacia catechu*	Fabaceae	HIV-1	(88)
	*Acacia catechu*	Fabaceae	HIV-1	(88)
	*Quercus persica*	Fagaceae	HSV-I	(90)
	*Geranium thunbergii*	Geraniaceae	Influenza virus, H1N1, H3N2, influenza type B	(91)
	*Pelargonium sidoides*	Geraniaceae	HIV-1	(92)
	*Ribes nigrum*	Grossulariaceae	Influenza A virus	(94)
	*Hamamelis virginiana*	Hamamelidaceae	Influenza A virus and human papillomavirus	(95)
	*Prunella vulgaris*	Lamiaceae	Lentivirus	(101)
	*Scutellaria baicalensis*	Lamiaceae	RSV, HIV, Influenza, and Dengue viruses	(104)
	*Strychnos pseudoquina*	Loganiaceae	HSV-1 (KOS strain) and HSV-2 (333 strain)	(108)
	*Punica granatum*	Lythraceae	HSV-2	(109)
	*Magnolia officinalis*	Magnoliaceae	Dengue virus type 2	(111)
	*Cissampelos pareira Linn*	Menispermaceae	Dengue virus types 1-4 (DENV-1-4)	(113)
	*Ficus benjamina*	Moraceae	HSV-1 and HSV-2), varicella zoster virus (VZV	(114)
	*Ficus carica*	Moraceae	HSV-1, HSV-1, ECV-11, and ADV, influenza virus	(115)
	*Ficus religiosa*	Moraceae	HSV-2	(116)
	*Syzygium aromaticum L*.	Myrtaceae	HSV and HCV	(119)
	*Paulownia tomentosa*	Paulowniaceae	SARS-CoV papain-like protease (PLpro)	(123)
	*Phyllanthus amarus*	Phyllanthaceae	Acyclovir-resistant HSV strains, hepatitis B virus (HBV), HCV, and HIV	(126)
Polyphenols	*Phyllanthus niruri*	Phyllanthaceae	Acyclovir-resistant HSV strains, hepatitis B virus (HBV), HCV, HIV	(126)
	*Phyllanthus urinaria*	Phyllanthaceae	Acyclovir-resistant HSV strains, hepatitis B virus (HBV), HCV and HIV	(126)
	*Phyllanthus watsonii*	Phyllanthaceae	Acyclovir-resistant HSV strains, hepatitis B virus (HBV), HCV, and HIV	(126)
	*Limonium sinense*	Plumbaginaceae	HCV	(128)
	*Plumbago indica*	Plumbaginaceae	Influenza A (H1N1)	(129)
	*Agrimonia pilosa*	Rosaceae	Influenza viruses (H1N1 and H3N2)	(135)
	*Prunus dulcis*	Rosaceae	HSV-1	(136)
	*Pavetta tomentosa*	Rubiaceae	Dengue virus (DENV)	(138)
	*Aegle marmelos*	Rutaceae	Human coxsackieviruses B1-B6, rotavirus SA-11	(139)
	*Dimocarpus longan*	Sapindaceae	HCV (genotype 2a strain JFH1)	(140)
	*Torreya nucifera*	Taxaceae	SARS-CoV 3CLpro	(144)
	*Viola diffusa*	Violaceae	Hepatitis B virus	(147)
	*Alpinia katsumadai*	Zingiberaceae	influenza virus type A	(148)
	*Illicium verum Hook. f*.	Schisandraceae	Grouper iridovirus infection (GIV)	(190)
	*Camellia sinensis*	Theaceae	HIV, HTLV-1, HCV, influenza, and HBV	(145, 146)
	*Ocimum sanctum*	Lamiaceae	Dengue virus serotype-1 (DENV-1)	(26, 99)
	*Achillea fragrantissima*	Asteraceae	Poliomyelitis-1 virus	(47, 48)
	*Ephedra alata*	Ephedraceae	HSV	(47, 77)
	*Tamarix nilotica*	Tamaricaceae	HSV	(47, 143)
	*Moringa peregrina*	Moringaceae	HSV	(47, 189)
	*Capparis sinaica*	Capparaceae	Avian influenza strain H5N1	(47, 64)
	*Ficus sycomorus*	Moraceae	HSV-1	(56, 118)
	*Balanites aegyptiaca*	Balanitaceae	VSV	(56, 57)
	*Terminalia mollis*	Combretaceae	HSV-0	(56, 73)
	*Tuberaria lignosa*	Cistaceae	HIV	(70, 71)
	*Anthemis hyaline*	Asreraceae	SARS-CoV	(152)
	*Alnus japonica*	Betulaceae	SARS-CoV	(59)
	*Cassia tora*	Fabaceae	SARS-CoV	(156)
	*Psoralea corylifolia*	Fabaceae	SARS-CoV	(150)
	*Taxillus chinensis*	Loranthaceae	SARS-CoV	(268)
Polyphenols	*Citrus sinensis*	Rutaceae	SARS-CoV	(152)
	*Polygonum multiflorum*	Polygonaceae	SARS-CoV	(158)
	*Rheum officinale*	Polygonaceae	SARS-CoV	(158)
	*Rheum palmatum*	Polygonaceae	SARS-CoV	(159)
	*Citrus sinensis*	Rutaceae	SARS-CoV	(152)
Alkaloids	*Sambucus nigra*	Adoxaceae	Dengue virus serotype-2 (DENV-2)	(29)
	*Iresine Herbstii*	Amaranthaceae	Newcastle disease virus (NDV)	(31)
	*Combretum adenogonium*	Combretaceae	HIV-1	(72)
	*Cyperus rotundus*	Cyperaceae	HSV-1, HBV	(76)
	*Equisetum giganteum*	Equisetaceae	HSV-2	(78)
	*Cissampelos pareira Linn*	Menispermaceae	Dengue virus types 1-4 (DENV-1-4)	(113)
	*Ficus religiosa*	Moraceae	HSV-2	(116)
	*Phyllanthus amarus*	Phyllanthaceae	HCV	(125)
	*Plumbago indica*	Plumbaginaceae	Influenza A (H1N1)	(129)
	*Pavetta tomentosa*	Rubiaceae	Dengue virus (DENV)	(138)
	*Tarenna asiatica*	Rubiaceae	Dengue virus (DENV)	(138)
	*Moringa peregrina*	Moringaceae	HSV	(47, 189)
	*Capparis sinaica*	Capparaceae	HSV	(47, 65)
	*Balanites aegyptiaca*	Balanitaceae	VSV	(56, 57)
	*Lycoris radiata*	Amaryllis	SARS-CoV	(151)
	*Acanthopanacis cortex*	Araliaceae	SARS-CoV	(134)
Saponins	*Iresine Herbstii*	Amaranthaceae	Newcastle disease virus (NDV)	(31)
	*Anacardium occidentale*	Anacardiaceae	Simian (SA-11) virus	(33)
	*Panax ginseng*	Araliaceae	RSV	(41)
	*Panax ginseng*	Araliaceae	Murine norovirus (MNV) and feline calicivirus (FCV)	(42)
	*Balanites aegyptiaca*	Balanitaceae	VSV	(56, 57)
	*Capparis sinaica*	Capparaceae	HSV	(47, 65)
	*Combretum adenogonium*	Combretaceae	HIV-1	(72)
	*Vachellia nilotica*	Fabaceae	HSV-2	(87)
	*Lindernia crustacea*	Linderniaceae	Epstein–Barr virus (EBV)	(107)
	*Artocarpus integrifolia*	Moraceae	(SA-11) and human (HCR3) rotaviruses	(33)
	*Ficus religiosa*	Moraceae	HSV-2	(116)
Saponins	*Ficus sycomorus*	Moraceae	HSV-1	(56, 118)
	*Moringa peregrina*	Moringaceae	HSV	(47, 189)
	*Plumbago indica*	Plumbaginaceae	Influenza A (H1N1)	(129)
	*Pavetta tomentosa*	Rubiaceae	Dengue virus (DENV)	(138)
	*Tarenna asiatica*	Rubiaceae	Dengue virus (DENV)	(138)
Terpenoids	*Andrographis paniculata*	Acanthaceae	Dengue virus serotype-1 (DENV-1)	(26)
	*Baccharis gaudichaudiana DC*	Asteraceae	Bovine viral diarrhea virus, HSV-1, Poliovirus type 2 (PV-2), and vesicular stomatitis virus (VSV)	(49)
	*Baccharis spicata (Lam.) Baill*	Asteraceae	Bovine viral diarrhea virus (BVD), HSV-1, poliovirus type 2 (PV-2), and vesicular stomatitis virus (VSV)	(49)
	*Taxodium distichum*	Cupressaceae	Influenza A and B viruses	(75)
	*Euphorbia hirta*	Euphorbiaceae	HIV-1, HIV-2, SIV mac 251	(80)
	*Jatropha multifida*	Euphorbiaceae	Influenza A H1N1 virus	(82)
	*Torreya nucifera*	Taxaceae	SARS-CoV 3CLpro	(144)
	*Agrimonia pilosa*	Rosaceae	Influenza viruses (H1N1 and H3N2)	(135)
	*Tripterygium regelii*	Celastraceae	SARS-CoV	(144)
	*Gentiana scabra*	Gentianaceae	SARS-CoV	(156)
Carbohydra-tes	*Panax ginseng*	*Araliaceae*	Human rotavirus	(33)
	*Panax notoginseng*	*Araliaceae*	Influenza A H1N1 virus	(43)
	*Equisetum giganteum*	*Equisetaceae*	HSV-2	(78)
	*Prunella vulgaris*	*Lamiaceae*	HSV-1 and HSV-2 antigens virus antigen in Vero cells	(100)
	*Prunellae Spica*	*Lamiaceae*	Herpes simplex virus (HSV)	(102)
	*Laminaria japonica*	*Laminariaceae*	RSV	(105)
	*Plumbago indica*	*Plumbaginaceae*	Influenza A (H1N1)	(129)
	*Ardisia chinensis Benth*	*Primulaceae*	Coxsackie B3 Virus	(131)
	*Capparis sinaica*	*Capparaceae*	HSV	(47, 65)
	*Balanites aegyptiaca*	*Balanitaceae*	VSV	(56, 57)
	*Carissa edulis*	*Apocynaceae*	herpes simplex virus, chickenpox, and shingles	(38)

Alkaloids are another class of natural organic compounds which are classified into several groups based on their heterocyclic ring, such as tropanes, pyrrolidines, isoquinoline purines, imidazoles, quinolizidines, indoles, piperidines, and pyrrolizidines (278). Alkaloids are very promising against HIV-1, HSV-1, HSV-2, DNV, VSV, Influenza virus, and Newcastle disease virus (NDV) (Table 4). Different kinds of alkaloids showed anti-SARS activity including emetine, Ipecac, Macetaxime, tylophorine, and 7-methoxy cryptopleurine, through the inhibition of protease enzyme, RNA synthesis, and protein synthesis (244, 279). In addition, some alkaloids act against SARS CoV as a nucleic acid intercalating agent such as tetrandrine, fangchinoline, cepharanthine, and lycorine through degrading nucleic acids and inhibiting spike and nucleocapsid proteins (280). Virtual screening analysis revealed that 10-Hydroxyusambarensine and Cryptoquindoline—two alkaloid compound isolated from African medicinal plants showed anti-SARS CoV and anti-SARS CoV-2 activity through inhibition of their M_pro_ (256). Chloroquine, a derivative of alkaloid, is found to be active against anti-SARS CoV-2 (281). So, some PSMs as alkaloids can be alternative drug targets for COVID-19 (280).

Another class of PSMs, saponins (amphipathic glycosides), are found ubiquitously in plants which showed antiviral activities against Newcastle disease virus (NDV), Simian (SA-11) virus, Murine norovirus (MNV) and Feline calicivirus (FCV), RSV, VSV, HSV-1,HSv-2, HIV-1, Epstein–Barr virus (EBV), (SA-11) and human (HCR3) rotaviruses, Influenza virus, and Dengue virus (Table 4). Plants produce five carbon isoprene derived terpenes which are the largest and most diverse group of PSM. They are classified by monoterpenes, diterpenes, triterpenes, sesterterpenes, hemi terpenes, and sesquiterpenes (282). They exhibited antiviral activity against Bovine viral diarrhea virus, HSV-1, Poliovirus type 2 (PV-2) and vesicular stomatitis virus (VSV), Dengue virus serotype-1 (DENV-1), Influenza A and B viruses, HIV-1, HIV-2, SIV mac 251, and SARS-CoV (Table 4). Ten diterpenes, two sesquiterpenes, and two triterpenes showed anti-SARS activity with IC_50_ of 3–10 μM (283). *In silico* analysis also revealed that terpene Ginkgolide A can strongly inhibit SARS CoV-2 protease enzyme (284). Carbohydrates, mainly classified as monosaccharides, disaccharides, polysaccharides, and oligosaccharides (282), are found as antiviral agent against Human rotavirus, Influenza A virus, HSV-1, HSV-2, Herpes simplex virus (HSV), RSV, Coxsackie B3 Virus, and VSV [(285); Table 4]. Acyclovir is an FDA (Food and Drug Administration) approved antiviral drug which is obtained from *Carissa edulis* (Supplementary Table 1). It is mainly used for herpes simplex virus, chickenpox, and shingles. The group basis structure of some major compounds can be found in Table 5.

**Table 5:** Structures of some major PSMs and Drugs used against SARS CoV-2.

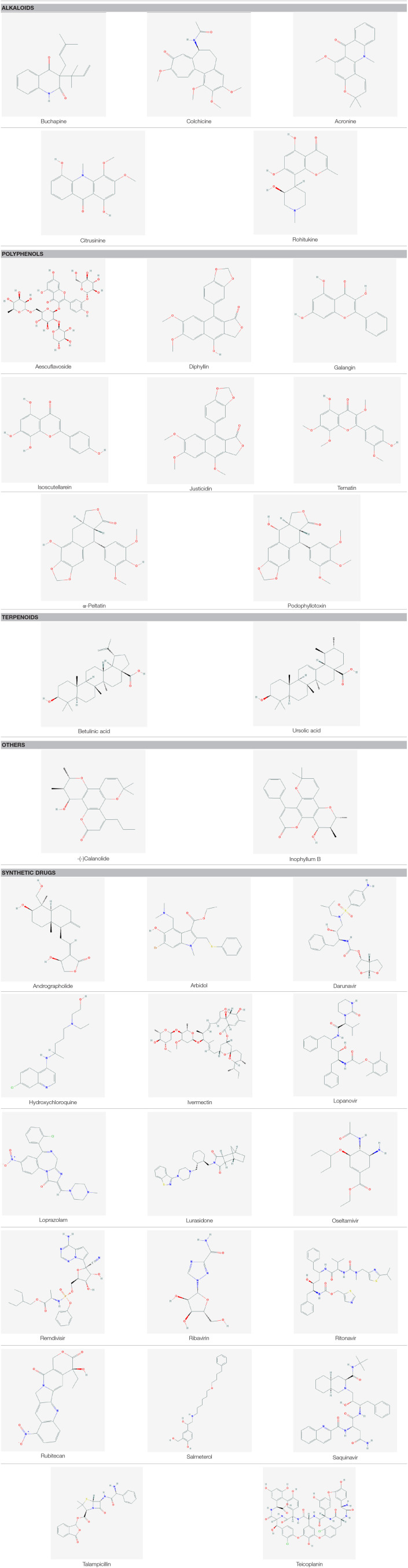

## Drug Discovery From PSMs: Addressing the Major Challenges Toward Future Insights

**Figure 2:**
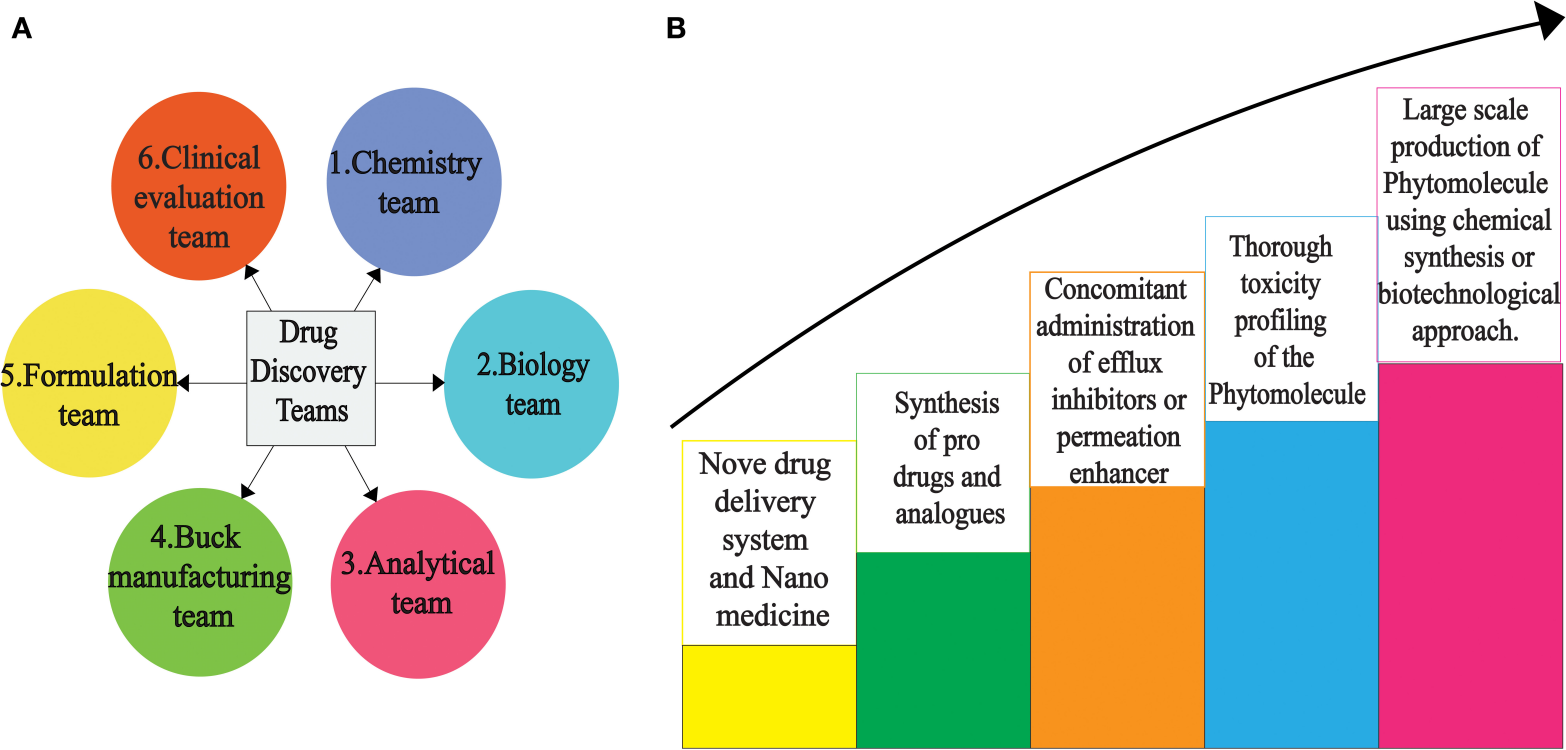
Scientific teams **(A)** to overcome various hurdles for successful novel drug discovery **(B)** from PSMs.

**Figure 3:**
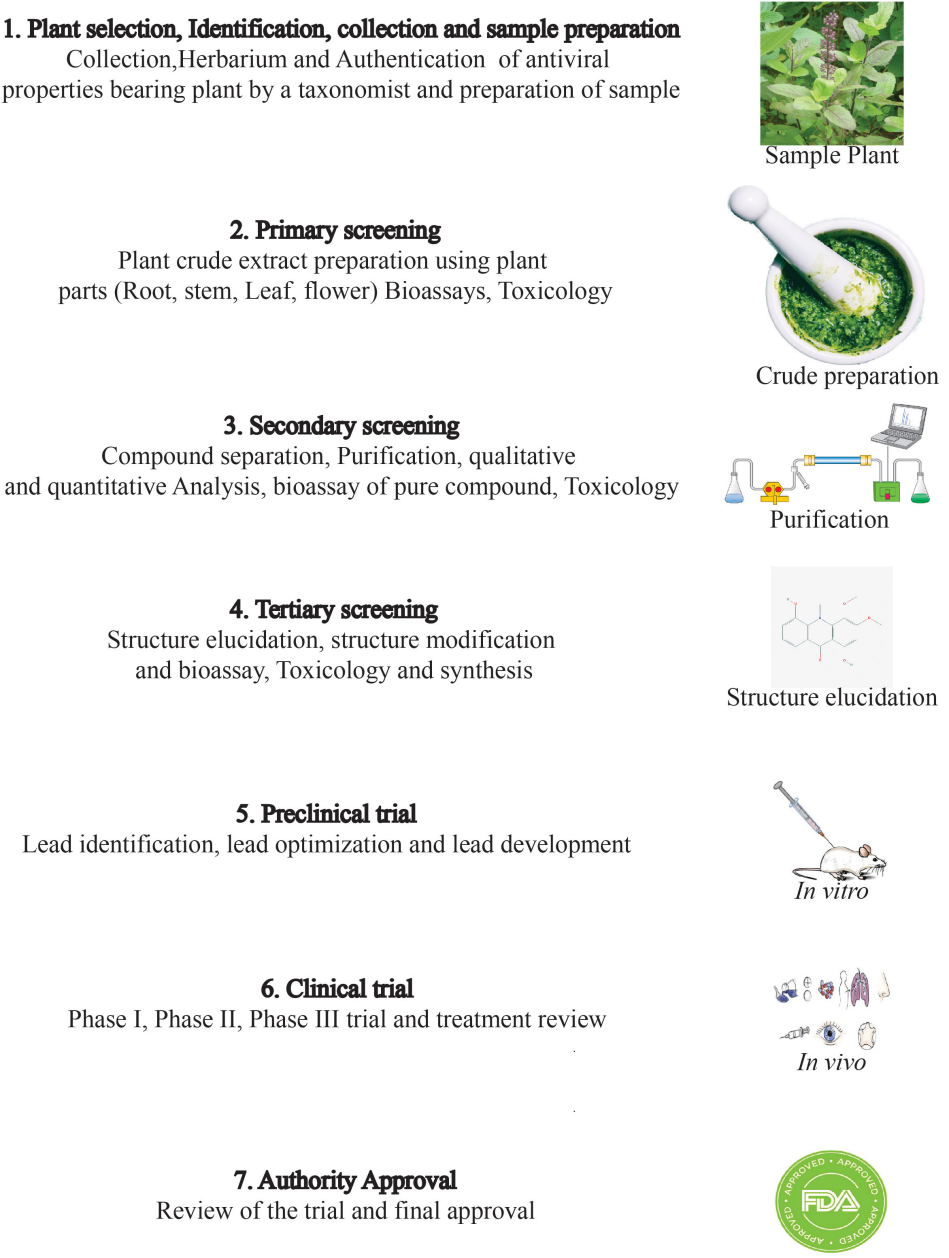
Various steps involved in the tedious drug discovery process from plant sources.

Drug discovery from plant metabolites refers to the extraction and purification of active ingredients from conventional cures. Natural plant products comprise complicated chemical structures which differ according to their numerous species. There are several classes of PSMs which are responsible for the biological activities of herbal medicines. PSMs exert their actions on molecular targets that differ from one case to the other. These targets may be enzymes, mediators, transcription factors, or even nucleic acids (286). Good knowledge of the chemical composition of plants leads to a better understanding of their possible and specific medicinal value. Drug discovery and development have become a wide interdisciplinary field over recent decades and many factors are involved in the successful evolution from a bioactive compound into a potential drug [(287, 288); Figure 2]. When existing methods with advanced technologies are applied, it can lead to a modern revelation of drugs, benefitting medicinal purposes (223, 289). The development of modern technologies has streamlined the screening of natural products in discovering new drugs. Research for drug discovery must create robust and prudent lead molecules, which is progressed from a screening hit to a drug candidate through structural elucidation and structure recognizable proof available from high throughput technology like GC–MS, NMR, IR, HPLC, and HPTLC. Utilizing these advanced technologies gives us an opportunity to perform research in screening novel molecules employing a computer program and database to set up common items as a major source for drug discovery. It finally leads to lead structure discovery. Powerful new technologies are revolutionizing natural herbal drug discovery (223). Steps associated with the drug discovery process from natural resources is illustrated (Figure 3).

However, several factors involving the conversion of a desirable compound into a valuable drug candidate include availability, bioavailability, intellectual property, and the strong pharmacokinetic profile of the compound (268, 290). Sometimes researchers find great bioactivity of a plant-derived compound in *in vitro* analysis but unfortunately, the desired compound becomes ineffectual under *in vivo* conditions (291). *In vivo* is a very crucial step to move to animal trials or subsequent clinical trials. Even if the compound shows promising activity in *in vivo* assay but it can still become ineffective in animal model trials due to a poor pharmacokinetic profile (292). Under *in vivo* condition, the target compound remains in direct contact with cells, while in animal models the compound moves to various stages where it might lose its bioactivity (292). For example, despite curcumin having promising antioxidant, anticancer, anti-inflammatory, and antimicrobial activities, it has not been released as a drug yet due to its poor bioavailability (292). Another propitious drug candidate, epigallocatechin gallate (EGCG), showed antioxidant, antihypertensive, anticancer, antimicrobial, and anti-inflammatory activity (293, 294) but unfortunately, it has also failed to obtain drug designation due to the same reason mentioned for curcumin (292).

To remedy these problems, researchers around the world are working to develop new approaches. Changing the administration route might increase the bioavailability of a compound. For example, the bioavailability of an anti-inflammatory compound, andrographolide, is increased when it is administered intravenously instead of through oral administration (295). Other methods to enhance the bioavailability of target compound include using drug delivery systems, the nano-formulation of a drug, using adjuvant systems, or altering structural analogs (220, 296). Furthermore, the modification of pharmacokinetic profiles of compounds like absorption, distribution, metabolism, and excretion can escalate its probability as drug candidate (268). Indeed, there is an urgent need for specific protocols for invention of novel bioactive compounds and for this purpose it is very crucial for related organizations, companies, and agencies, including the World Health Organization (WHO), Food and Drug Administration (FDA), European Medicines Agency (EMA), World Trade Organization (WTO), International Conference on Harmonization (ICH), World Intellectual Property Organization (WIPO), biotech companies, pharmaceutical pharmaceuticals companies, and several other companies and agencies, to work together. However, plant-originated therapeutics need to be taken under consideration against SARS-CoV-2 as they have already shown promising hopes for different critical conditions caused by deadly pathogens.

The seven major drug targets of SARS CoV-2 were described before (176). Similarly, screening of PSMs for drug establishment by molecular docking is efficient in terms of time and cost. Even the development of vaccines through computational biology was found to be effective for previous severe viruses like MERS using animal models, target antigens, and probable vaccine candidates (181). But still, there exists a lack of a complete review for PSMs as alternative drug therapeutics. Our review aims at establishing PSMs as a strong and safe candidate for the treatment of SARS CoV-2. Through suggesting probable antiviral plant metabolites or screening, druggability analysis of plant metabolites against SARS-CoV-2 has become a time-saving practice (280, 297). Without establishing a drug development pipeline that includes clinical trials, these suggested candidate PSMs will end up only in journal publications or be shelved as herbal formulations on a supermarket store as a traditional medicine and will never be a modern drug. Undoubtedly, the plant an underutilized source of novel bioactive compounds and is one of the hotspots to fight against this microbial resistance war. The decrypting of PSMs is not increasing so much in comparison to the number of metabolites produced from plants. A biotechnological approach can offer a desired amount of secondary metabolites in a rapid and eco-friendly way against SARS-CoV-2 (298). In addition, plant metabolomics are now used as a tool for discovery of novel drugs from plant resources (299). Characterization of genes and proteins involved in secondary metabolic pathways are also very crucial to understand. Therefore, omics approaches (transcriptomics, proteomics, and metabolomics) have paramount importance in food research and drug discovery (300, 301) for human welfare. Genetic modifications for engineering plant metabolites can be helpful for reaching a specific drug. Quality control of natural products is also very important. So, laboratory support, skilled manpower, and funding is also very important for drug discovery from natural resources.

## Conclusions

Scientists all around the world are trying to discover the most effective antiviral drug to combat SARS CoV-2. In this situation, our study accentuated some plant secondary metabolites that showed prominent antiviral activity against coronaviruses through impeding the main machinery used in their pathogenesis and replication cycle. The *in vitro, in vivo*, and *in silico* investigations revealed numerous plant-derived compounds with promising anti- SARS CoV and anti- SARS CoV-2 activity [Table 2; (179, 219, 222, 233–261, 297)]. Plants are a dramatically underutilized source of bioactive compounds with a broad spectrum of antiviral activities. Some Chinese traditional plant formulations have been reported as being anti- SARS CoV-2 and this formulation is also provided in COVID patients (302, 303). We reported here on 219 plants which act against a wide range of DNA/RNA viruses, but the plant PSMs that showed promising activity against SARS CoV and MERS might be a desired drug candidate against SARS CoV-2. So, this review gathered all antiviral plants in a single platform to facilitate laboratory-based research for the development of novel drug/molecular therapeutics to overcome this and future pandemic situations. The world is facing a serious health crisis, and it needs an effective solution to combat the burning flame of COVID-19. Researchers are trying to find an effective way to overcome this situation, and the present study could help them to think with a new dimension by using the knowledge from the databases based on the plant metabolites (304, 305). Finally, advanced and rapid acting extraction, purification, and characterization techniques used for plant metabolites as well as multidisciplinary expertise and funding are very essential for novel drug discovery.

## Literature Study

Articles were selected and identified by searching specific keywords and journal citations for each section of a manuscript. Related peer reviewed scientific journal articles were screened from different journal depositories after reviewing abstracts and original data.

## Author's Note

The authors initiated this project to facilitate the research on molecular therapeutics from plant sources as an immediate action in response to the COVID-19 pandemic situation.

## Author Contributions

FB and MH designed the project. FB prepared the first draft. FB, SH, TR, and MH have investigated the data and completed the manuscript. All authors have read through the manuscript and approved it for submission and publication.

## Conflict of Interest

The authors declare that the research was conducted in the absence of any commercial or financial relationships that could be construed as a potential conflict of interest.

## Supplementary Material

Supplementary Figure 1 Different plant families showing antiviral properties. (Each portion of the pie chart describes a specific Family alongside its total number of plants that have antiviral properties).

Supplementary Table 1 List of secondary metabolites found from medicinal plants.
