# Supplementary figures and images for: Plants Metabolites: Possibility of Natural Therapeutics Against the COVID-19 Pandemic
Source: Front Med (Lausanne). 2020 Aug 7;7:444. doi: 10.3389/fmed.2020.00444 (PMC7427128; doi:10.3389/fmed.2020.00444)

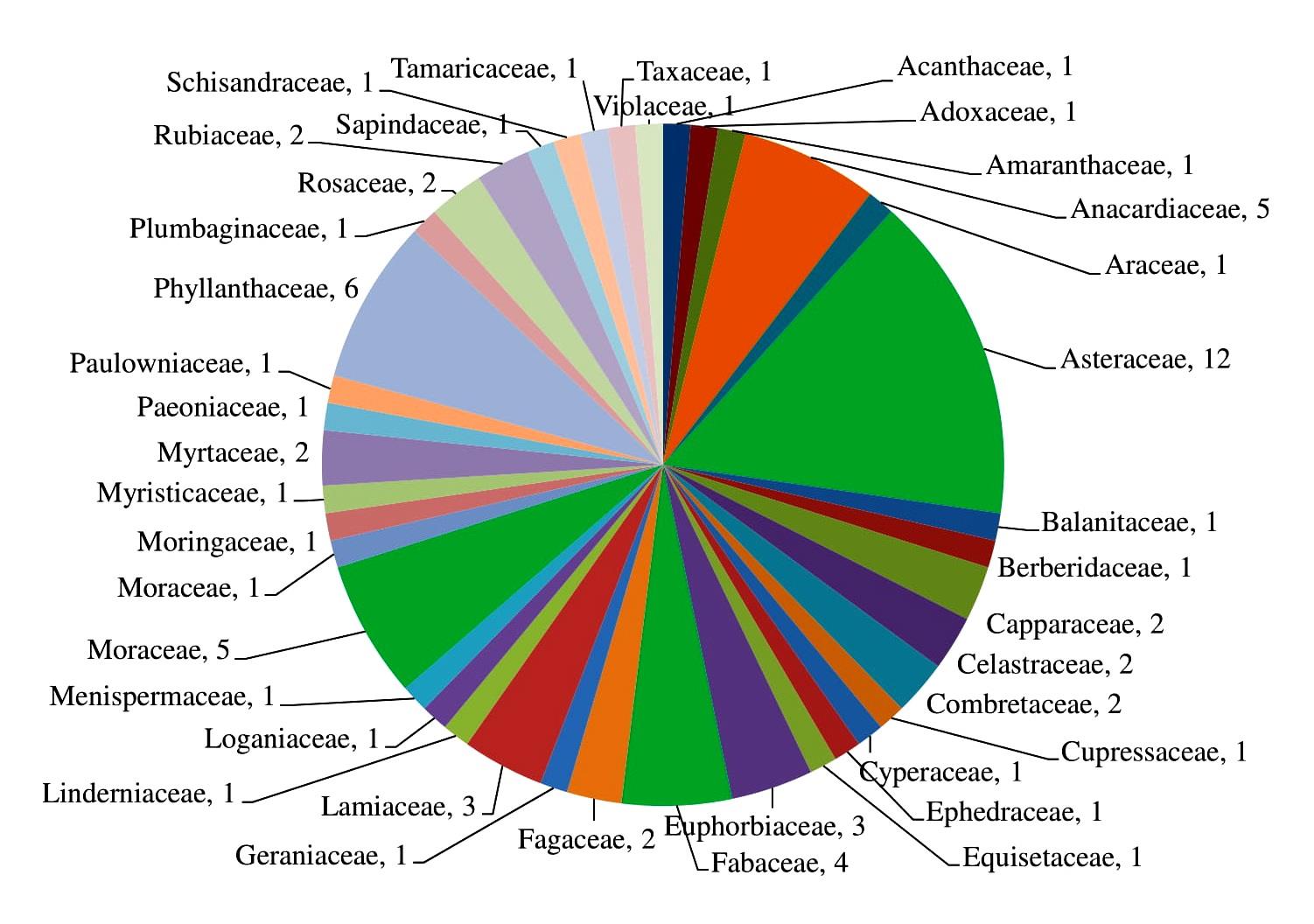

Supplement: Supplementary Figure 1 — Different plant families showing antiviral properties. (Each portion of the pie chart describes a specific Family alongside its total number of plants that have antiviral properties). [file Image_1.jpeg]
