# Supplementary material for: Plants Metabolites: Possibility of Natural Therapeutics Against the COVID-19 Pandemic
Source: Front Med (Lausanne). 2020 Aug 7;7:444. doi: 10.3389/fmed.2020.00444 (PMC7427128; doi:10.3389/fmed.2020.00444)
Supplement: Supplementary Table 1 — List of secondary metabolites found from medicinal plants. [file Table_1.doc]

| **Serial No**  **Supplementary Table 1.** List of secondary metabolites found from medicinal plants | **Compound** | **Plant Source** | **Plant Parts** | **Family** | **Susceptible Virus** | **Mode of Action** | **Status of the Antiviral Assay** | **Ref** |
| --- | --- | --- | --- | --- | --- | --- | --- | --- |
|  | Diterpneoid | *Andrographis paniculata* | Arial parts | Acanthaceae | Dengue virus serotype-1 (DENV-1) | NR | *In vitro* | 26 |
|  | Flavonoid | *Avicennia marina* | Leaf |  | Human immunodeficiency virus (HIV) and Herpis simplex virus (HSV) | Active flavonoid compoundspenetrated in the target cell and HSV (KOS) replication was inhibited | *In vitro* | 27 |
|  | Aurantiamide acetate | *Baphicacanthus cusia* | Root |  | Influenza A virus | NF-κB signaling pathway inhibited | *In vitro* and *In vivo* | 28 |
|  | Alkaloids, flavonoids and coumarins | *Sambucus nigra* | Flower and Leaf | Adoxaceae | Dengue virus serotype-2 (DENV-2) | NR | *In vitro* | 29 |
|  | Polyphenol derivatives, Lectins | *Sambucus nigra* | Fruit |  | Infectious bronchitis virus (IBV) - chicken coronavirus | Lectins block the access to host-cell receptors used by IBV | *In vitro* | 30 |
|  | Alkaloids, Anthraquinones, Glycosides, Flavonoids, Saponins, Phenols, Terpenoids, Sugar bearing compound, Protein, Thiols and Inferences | *Iresine Herbstii* | Leaf and Stem | Amaryllidaceae | Newcastle disease virus (NDV) | NR | *In vivo* | 31 |
|  | Lycorine, Homolycorine and 2-O-acetyllycorine | *Leucojum vernum* | Bulb |  | HIV-1 | Extract showed HIV-1 replication inhibitory activity on MT4 cells | *In vitro* | 32 |
|  | Tannins, Flavonoids, Terpenes and Saponines | *Anacardium occidentale* | Leaf | Anacardiaceae | Simian (SA-11) virus | NR | *In vitro* | 33 |
|  | Tannins, Gallic acid, Flavonoids like quercetin and quercitrin, Phenolics, Triterpenes | *Rhus aromatica* | Bark |  | HSV-1 and HSV-2 | Fragrant sumac extract interferes with virion envelope structures/masking the viral compounds which are vital for adsorption/entry into the host cells | *In vitro* | 34 |
|  | Gallic acid, quercetin, Kampferol, Glycosides | *Rhus parviflora* | Leaf |  | HIV-1 | Anti-HIV-1 property of extract might inhibited the protease activity of HIV-1 | *In vitro* | 35 |
|  | Tannins (Prodelphinidin and procyanidin units) | *Schinus terebinthifolia* | Bark of Stem |  | HSV-1 | Inhibited the HSV 1 strains *In vitro* and found effective at the attachment and penetration stages, and showed virucidal activity (confirmed by transmission electron microscopy). | *In vitro* and *In vivo* | 36 |
|  | Tannins and Flavonoids | *Spondias lutea* | Bark | Anacardiaceae | Human (HCR3) rotaviruses | Inhibited the human rotavirus cell propagation | *In vitro* | 33 |
|  | Flavonoids | *Spondias lutea L.* | Leaf |  | Simian (SA-11) and human (HCR3) rotaviruses | Inhibited the propagation of rotavirus and protected the cell cultures against virus invasion | *In vitro* | 33 |
|  | Chalcones and Coumarins | *Angelica keiskei* | Leaf | Apiaceae | Severe acute respiratory syndrome coronavirus (SARS-CoV) | Xanthoangelol E has specific inhibitory activity against cysteine protease specifically using the cell-based 3CLpro cis-cleavage assay | *In vitro* and *In silico* | 37 |
|  | Acyclovir | *Carissa edulis* | Root | Apocynaceae | HSV | Inhibited the plaques formation in Vero E6 cells infected with wild type strains of HSV (7401H HSV-1 & Ito-1262 HSV-2) or resistant strains of HSV (TK- 7401H HSV-1 & APr 7401H HSV-1) | *In vitro and In vivo* | 38 |
|  | 3-OH-4-OMeAld and 2-OH-4-OMeAld | *Hemidesmus indicus* | Root |  | HSV-1 and 2 HSV-2 | Inhibited the early step of HSV- 1 and HSV-2 replication by its anti-ER α-glucosidase inhibitory activity | *In vitro* | 39 |
|  | Apigenin and Luteolin | *Arisaema tortuosum* | Leaf | Araceae | Acyclovir-resistant HSV-2 and HSV-1 | Inhibit multiple cycles of viral replication and limit the production of infectious virus particles | *In silico* | 40 |
|  | Ginsenosides, Saponins | *Panax ginseng* | Root | Araliaceae | RSV | In vitro: By suppressing the expression of RSV-induced inflammatory cytokine genes (IL-6 and IL-8) and the formation of reactive oxygen species (ROS) in epithelial cell cultures  In vivo: By enhancing the level of interferon-γ (IFN-γ) producing dendritic cells subsequent to RSV infection | *In vitro* and *In vivo* | 41 |
|  | Ginsenosides, saponins | *Panax ginseng* | Root |  | Murine norovirus (MNV) and Feline calicivirus (FCV) | NR |  | 42 |
|  | Epigallocatechin gallate, Theaflavin digallate, Genistein, Hesperidin, Polysaccharides | *Panax ginseng* | NR |  | Human rotavirus | NR | *In vitro* | 33 |
|  | Ginsenosides, Polysaccharides, and Essential oils | *Panax notoginseng* | Root |  | Influenza A H1N1 virus | Enhancing antiviral interferon-mediated immune responses and natural Killer cell activity | *In vitro* and *In vivo* | 43 |
|  | Biaron C | *Aloe arborescens* | Leaf | Asphodelaceae | Influenza A and influenza B viruses | Impired the replication of influenza A (H1N1 and H3N2), influenza B viruses (Yamagatal and Beiying), human rhinovirus and coxsackievirus was significantly reduced | *In vitro* | 44 |
|  | AG is Anthraquinones, Mannose-6-phosphate, Gluco-mannans | *Aloe vera* | Leaf |  | HSV-2 | By blocking virus adsorption, attachment or entry to the host cell through disrupting the viruse envelope | *In vitro* | 45, 46 |
|  | Phenolic acids, Flavonoids (apigenin, apigeninglucoside, luteolin, cirsiliol, diosmetin), Lignans, Terpenic lactones and Alkamides | *Achillea fragrantissima* | Aerial part | Asteraceae | Poliomyelitis-1 virus | NR | *In vitro* | 47, 48 |
|  | Flavonoids, Clerodane Diterpenoids, Phenolics, Hydroxycinnamic acids | *Baccharis gaudichaudiana DC* | NR |  | Bovine viral diarrhea virus, HSV-1, Poliovirus type 2 (PV-2) and vesicular stomatitis virus (VSV) | NR | NR | 49 |
|  | Diterpenoids | *Baccharis spicata (Lam.) Baill* | NR |  | Bovine viral diarrhea virus (BVD), HSV-1, poliovirus type 2 (PV-2) and vesicular stomatitis virus (VSV) | NR | NR | 49 |
|  | Triterpenoids, Steroids | *Bidens subalternans DC* | NR |  | Bovine viral diarrhea virus, HSV-1, poliovirus type 2 (PV-2) and vesicular stomatitis virus | NR | NR | 49 |
|  | Flavonoid glycosides and Caffeoyl quinic acids | *Eupatorium perfoliatum* | Aerial parts |  | Influenza A virus (IAV) H1N1 | blocked the attachment of IAV and inhibited with virus-induced hemagglutination | *In vitro* | 50 |
|  | Flavonoids and Terpenes | *Jasonia montana* | Aerial part |  | Poliomyelitis-1 virus | NR | *In vitro* | 47 |
|  | Phenylpropanoids, Flavonoids, Essential oils, Polyphenols, Tannins, Triterpenes | *Pluchea sagittalis (Lam.) Cabrera* | NR |  | Bovine viral diarrhea virus (BVDV) (HSV-1), poliovirus type 2 (PV-2) and vesicular stomatitis virus (VSV) | NR | NR | 49 |
|  | Silymarin, Quercetin and Kaempferol | *Silybum marianum* | NR |  | Chikungunya virus (CHIKV), Hepatitis C virus (HCV) | Inhibited the entry of HCV, synthesis of RNA, expression of viral protein | *clinical presentations* | 51 |
|  | Terpenoids, Flavonoids, Essential oils | *Tagetes minuta L* | NR | Asteraceae | Bovine viral diarrhea virus, HSV-1, poliovirus type 2 (PV-2) and vesicular stomatitis virus | NR | NR | 49 |
|  | Phenolic acids (chlorogenic acids) and Sesquiterpene lactones (parthenolide) | *Tanacetum parthenium* | Aerial part |  | HSV-1 | NR | *In vitro* and *In vivo* | 52 |
|  | Flavonoids, D-glucopyranoside, Quercetin, Luteolin | *Taraxacum officinale* | Leaf |  | HCV | Direct inhibition of viral enzymes by suppression of HCV RNA level and NS5B gene expression | *In-vitro* and *In silico* | 53 |
|  | Polyphenolic compound (epigallocatechin-gallate) | *Taraxacum officinale* | Herb |  | Influenza virus type A, H1N1. | By inhibiting polymerase activity and reduced virus nucleoprotein (NP)/ RNA level | *In vitro* | 54 |
|  | Flavonoids (Apigenin, quercetin, kaempferol, falcarinol, selinene, limonene and zerumbone) | *Tridax procumbers* | Stem |  | Dengue virus serotype-2 (DENV-2) | Reduces CPE in infected cells evidenced by the occurrence of round and spindle shaped cells | *In vitro* | 55 |
|  | Phenolics (gallic acid) | *Vernonia cinerea* | Leaf |  | Dengue virus serotype-2 (DENV-2) | Reduces CPE in infected cells evidenced by the occurrence of round and spindle shaped cells | *In vitro* | 55 |
|  | Carbohydrates, Lipids, Proteins, Alkaloids, Flavonoids, Saponins and Organic acids | *Balanites aegyptiaca* | Leaf | Balanitaceae | VSV | NR | *In vitro* | 56, 57 |
|  | Icariin and quercetin | *Epimedium koreanum Nakai* | Herb | Berberidaceae | Porcine epidermic diarrhea virus (PEDV) | Inhibited the cytopathic effect by porcine epidemic diarrhea (PED) and viral replication in vero cells | *In vitro* and *In vivo* | 58 |
|  | Diarylheptanoids | *Alnus japonica* | Bark | Betulaceae | SARS-CoV | Hirsutenone inhibited papain-like protease of SARS Coronavirus catechol and α,β-unsaturated carbonyl moiety in the molecule needed to inhibit cysteine protease of SARS-CoV | *In vitro* | 59 |
|  | Diarylheptanoids | *Alnus japonica* | Bark |  | Influenza virus KBNP-0028 (H9N2) | platyphyllone and platyphyllonol-5-xylopyranoside inhibits replication of the virus | *In vitro* | 60 |
|  | Indigo, Sinigrin, Aloeemodin and Hesperetin | *Isatis indigotica* | Root | Brassicaceae | SARS coronavirus | In cell-free and cell-based assays, inhibited the cleavage activity of the SARS coronavirus 3C-like protease | *In vitro* | 61 |
|  | Polyphenolic compound | *Canarium album (Lour.)* | Fruit (Fresh) | Burseraceae | Influenza A virus (IAV) | By nhibiting neuraminidase activity of the virus | *In vitro* and *In silico* | 62 |
|  | Apetalic acid and Calanolides | *Calophyllum brasiliense* | Leaf | Calophyllaceae | HIV-1 | NR | *In vitro and In vivo* | 63 |
|  | Flavonoids (quercetin , isoquercetin and rutin) | *Capparis sinaica* | Whole plant | Capparaceae | Avian influenza strain H5N1 | The antiviral activity of quercetin and rutin inhibits the replication of both influenza A and B viruses | *In vitro* | 47, 64 |
|  | Tannins, Flavonoids, Carbohydrates and/or Glycosides, Resins, Sterol, Saponins and Alkaloids | *Capparis sinaica* | Whole plant |  | HSV | NR | *In vitro* | 47, 65 |
|  | Flavanols and Hydroxycinnamic acid, Protein | *Capparis spinosa* | Seed |  | HIV-1, HSV-2 | Contains protein with potent antiproliferative activity toward tumor cells and inhibitory activity toward HIV-1 reverse transcriptase | *In vitro* | 66 |
|  | Natural lupane triterpenoids | *Cassine xylocarpa* | Stem | Celastraceae | HIV | Compounds displayed inhibitory effects on HIV-1 replication | *In vitro* | 67 |
|  | Pentacyclic Lupane-Type Triterpenoids | *Maytenus cuzcoina* | Root bark |  | HIV | By inhibiting HIV-1 replication strongly | *In vitro* | 67 |
|  | Salacinol, Kotalanol, and Catechins | *Salacia reticulata* | Stem and Root |  | Influenza A virus H1N1 | In Murine lung tissues, reduces H1N1 influenza clinical symptoms due to enhanced natural Killer cell activity | *In vitro* and *In vivo* | 68 |
|  | Polyphenols | *Cistus incanus* | Whole plant (Fresh) | Cistaceae | HIV (clinical HIV-1 and HIV-2 ) and Filoviruses, Ebola and Marburg virus | By targetting the viral envelope proteins | *Clinically used* | 69 |
|  | Ellagitannins and Ellagic acid derivative | *Tuberaria lignosa* | Whole plant |  | HIV | By inhibiting the entry | *In vitro* | 70, 71 |
|  | Flavonoids, Terpenoids, Alkaloids, Tannins, Glycosides and Saponins | *Combretum adenogonium* | Root and Stem bark | Combretaceae | HIV-1 | Glycosides in the root and stem bark extracts only could be speculated as the major contributing factor for the anti-HIV-1 protease inhibitory activity | *In vitro* | 72 |
|  | Triterpenes, Flavonoids, Ellagitannins | *Terminalia mollis* | Root and Bark |  | HSV-0 | NR | *In vitro* | 56, 73 |
|  | Hydrolysable tannins, Tellimagrandin I, iridoids | *Cornus canadensis* | Leaf | Cornaceae | HSV-1 | Hydro-ethanolic extract acts directly on HSV-1 and inhibits virus absorption by host cells | *In vitro* | 74 |
|  | Lignans, Diterpenes, Flavonoids, Proanthocyanidins, and Sterols | *Taxodium distichum* | Stem | Cupressaceae | Influenza A and B viruses | By targettings hemagglutinin and neuraminidase-related activities of influenza virus | *In vitro* | 75 |
|  | Monoterpenoids, Sesquiterpenoids, Triterpenoids, Sterols, Alkaloids, Flavonoids and Phenolic compounds | *Cyperus rotundus* | Rhizome | Cyperaceae | HSV-1, HBV | DNA replication inhibition | *In vitro* | 76 |
|  | Protocatecuic acid, Caffeic acid, Epicatechin, Rutin, Resveratrol, Quercitin, Kaempferol | *Ephedra alata* | Aerial part | Ephedraceae | HSV | NR | *In vitro* | 47, 77 |
|  | Isoflavonoid, Indoles, Phytoesterols, Polysaccharides, Sesquiterpenes, Alkaloids, Glucans, and Tannins | *Equisetum giganteum* | Root and Stem | Equisetaceae | HSV-2 | By interfering with viral cell attachment and entry though unable to block viral replication post entry | *In vitro* and *In vivo* | 78 |
|  | Triterpenes and Steroids | *Euphorbia denticulata* | Aerial part | Euphorbiaceae | HSV | NR | *In vitro* | 79 |
|  | Tannins, Diterpenes | *Euphorbia hirta* | Whole plant |  | HIV-1, HIV-2, SIV mac 251 | NR | *In vitro* | 80 |
|  | Carolignans, Lignans, Polyphenols | *Euphorbia sikkimensis* | Aerial parts |  | HIV-1 | NR | *In vitro* | 81 |
|  | Diterpenoids, Jatrophane-type diterpenoids, and Coumarino-type lignoids, Lathyrane-type diterpenoids, Multifidone, Multifidanol, and Multifidenol. | *Jatropha multifida* | Stem |  | Influenza A H1N1 virus | Inhibited viral binding to host cells surface, endocytosis, membrane fusion, or uncoating and by inhibiting replication by inhibiting viral RNA polymerase/NA activities | *In vitro* | 82 |
|  | Flavonoid and Polyphenol | *Acacia arabica* | Leaf | Fabaceae | Influenza A virus H9N2 | By blocking of HA protein of the virus, inhibits the H9N2 virus replication, prevention adsorption of viruses to cells | *In vitro* | 83 |
|  | Chlorogenic acid, Catechins, Proanthocyanidins and Resveratrol | *Arachis hypogaea* | Peanut skins |  | Human Influenza Viruses (type A and B) | By targeting different replication stages of the influenza virus | *In vitro* | 84 |
|  | Luteolin and Vitexin | *Aspalathus linearis* | Leaf |  | Rhesus rotavirus (RRV), Simian rotavirus (SA-11) infection | NR | *In vitro* | 85 |
|  | Apigenin-8-C-glucoside (vitexin) | *Erythrina speciosa* | Leaf |  | HSV-1 | vitexin accommodates in the HSV-1 binding pocket of thymidine kinase, HAV 3C proteinase, and Hepatitis B virus capsid protein | *In vitro* and *In silico* | 86 |
|  | 18ß-glycyrrhetinic acid | *Glycyrrhiza glabra L* | Root |  | Rhesus rotavirus (RRV) | NR | *In vitro* | 85 |
|  | Phenolic glycosides | *Senna angustifolia* | Leaf | Fabaceae | Dengue virus serotype-2 (DENV-2) | Reduces CPE (…..) in infected cells evidenced by the occurrence of round and spindle shaped cells | *In vitro* | 55 |
|  | Saponins and Flavonoids | *Vachellia nilotica* | Bark |  | HSV-2 | NR | *In vitro* | 87 |
|  | Catechin, Kaempferol, Quercetin, 3,4',7-trihydroxyl-3′,5-dimethoxyflavone, Rutin, Isorhamnetin, Epicatechin, Afzelechin, Epiafzelechin, Mesquitol, Ophioglonin, Aromadendrin and Phenol | *Acacia catechu* | Barks of stem |  | HIV-1 | HIV-1 replication suppresstion by viral protease and through trans-activator protein (Tat) inhibition | *In vitro* | 88 |
|  | Catechu | *Quercus brantii L Acorn.* | Fruit | Fagaceae | HSV-1 | Among the five fractions of ethanolic extract, only chloroform fraction showed high inhibitory effect against HSV- replication | *In vitro* | 89 |
|  | Flavonoids, Phenolics and Tannins | *Quercus persica* | Fruit |  | HSV-I | Forming a complex with the virusat binding sites on the cells, most probably by preventing the virus to attach to the cellular biding sites | *In vitro* | 90 |
|  | Phenolic, Flavonoid, and Flavonol compounds | *Quercus persica* | Fruit |  | HSV-2 | NR | *In vitro* | 90 |
|  | Gallic acid, Protocatechuic acid, Corilagin, Geraniin, Ellagic acid, Kaempferitrin, Kaempferol 7-O-rhamnoside, Quercetin, Kaempferol | *Geranium thunbergii* | Aerial part (Dried) | Geraniaceae | Influenza virus, (H1N1, H3N2, Influenza type B | Neuraminidase inhibition | NR | 91 |
|  | Polyphenolics | *Pelargonium sidoides* | Root |  | HIV-1 | Directly Interferes with viral infectivity follwed by blocking HIV-1 attachment to target cells resulted in virus entry protectition and targets HIV-1 envelope proteins though virus particles bearing the heterologous VSV-G protein instead of HIV-1 proteins in their envelopes are much less sensitive to inhibition by PS extract | *In vitro* | 92 |
|  | EPs® 7630 | *Pelargonium sidoides* | Root |  | Influenza A virus strains (H1N1, H3N2), RSV, Human coronavirus, Parainfluenza virus, and Coxsackie virus | NR | *In vitro* | 92 |
|  | Tannins (prodelphinidins and proanthocyanidins) phenolics and antioxidants (He et al., 2010; Tabart et al., 2011) | *Ribes nigrum* | Fruit | Grossulariaceae | Influenza A virus | Virucidal properties bearing extract blocks the virus replicationat at early step of infection and assume as similar anti-adhesive manner like CYSTUS052 (A polyphenol rich plant extract) | *In vitro* and *In vivo* | 94 |
|  | Hydrolysable tannins and Pseudotannins, Gallic acid, Epigallocatechin gallate or Hamamelitannin | *Hamamelis virginiana* | Leaf and Bark | Hamamelidaceae | Influenza A Virus and Human Papillomavirus | Receptor binding (but not neuraminidase) inhibition | *In vitro* | 95 |
|  | Ligomeric to polymeric proanthocyanidins (PA), Amamelitannin, Catechin | *Hamamelis virginiana* | Bark |  | HSV-1 | NR | NR | 96 |
|  | Rosmarinic acid (RA) | *Melissa officinalis* | Leaf and Stem (Dried) | Lamiaceae  Lamiaceae | Enterovirus 71 | Viral attachment and entry reduction, cleavage of eukaryotic translation initiation factor 4G (eIF4G), generation of reactive oxygen species (ROS), and translocation of heterogeneous nuclear ribonucleoprotein A1 (hnRNP A1) from nucleus to cytoplasm occured. It alleviated the activity of EV71-induced hyperphosphorylation of p38 kinase and EPS15 | *In vivo* | 97 |
|  | Caryophyllen , Citral, Citronellal, b-Cubeben, Menthylheptenon , Caryophyllenoxid, Ocimen | *Melissa officinalis* | Oil |  | Enveloped herpesviruses | Host cell adsorption and penetration is inhibited due to the effects of oil on envelop proteins thereby impaired their ability to infect host cells | *In vitro* | 98 |
|  | Flavonoids (orientin and vicenin) | *Ocimum sanctum* | Arial parts |  | Dengue virus serotype-1 (DENV-1) | Blocks the E protein of DENV-1, preventing it from entering the cells | *In vitro* | 26, 99 |
|  | Terpenoid and Polyphenol | *Ocimum sanctum* | Leaf |  | Influenza A virus H9N2 | Masking/blocking of HA protein of the virus, inhibition the H9N2 virus replication, prevention adsorption of viruses to cells | *In vitro* | 83 |
|  | Polysaccharide fraction | *Prunella vulgaris* | Spikes (dried) |  | HSV-1 and HSV-2 antigens virus antigen in Vero cells | Polysaccharides (heparin, dextran, carrageenans, pentosan polysulfate, fucoidan, and sulfated xylogalactans) acted as competitors of receptors (heparin sulfate) to the viral glycoproteins thereby impired the binding of virus to the host cell. | *In vitro* | 100 |
|  | Polyphenol, Carbohydrates | *Prunella vulgaris* | Plant and Seed |  | Lentivirus | Inhibition of lentivirus replication | *In vitro* | 101 |
|  | Polysaccharide PSP-2B | *Prunellae Spica* | Whole plant |  | Herpes simplex virus (HSV) | NR | *In vitro* | 102 |
|  | sulphated Carbohydrates | *Rosmarinus officinallis* | Whole plant |  | HSV-1 | HSV-1 replication inhibition | *In vitro* | 103 |
|  | Glycosidic derivative of apigenin apigenin-7-O-β-D-glucopyranoside | *Schizonepeta tenuifolia Briq.* | Lyophilized powder of herb |  | Enterovirus 71 (EV71) | Reduced viral attachment and entry, eukaryotic translation initiation factor 4G (eIF4G) cleavage by EV71 protease, 2Apro; virus-induced reactive oxygen species (ROS) formation; and relocation of heterogeneous nuclear ribonucleoprotein A1 (hnRNP A1) from the nucleus to the cytoplasm resulted in the prevention of the virus. | *In vitro* and *In vivo* | 97 |
|  | Baicalin, Flavonoids | *Scutellaria baicalensis* | Root |  | RSV, HIV, influenza, and Dengue viruses | Blocks RSV infection and reduces inflammatory cell infiltration and lung injury in mice | *In vivo* | 104 |
|  | Polysaccharide | *Laminaria japonica* | Whole plant | Laminariaceae | RSV | RSV replication inhibitin in a dose dependent manner | *In vitro* | 105 |
|  | Kaempferol | *Lilium candidum* | Stem, Leaf and Fruit | Liliaceae | HSV-1 and HSV-2 and Varicella-Zoster Virus (VZV) | By blocking adsorption of virus to the host cells | *In vitro* | 106 |
|  | Opuntin B, Triterpene Saponin, Seroids, and Phenylethanoids | *Lindernia crustacea* | Whole plant | Linderniaceae | Epstein–Barr virus (EBV) | Replication and transcriptional activator (Rta)expression inhibition in the viral lytic cycle | *In vitro* | 107 |
|  | Quercetin 3-O-methyl ether (3MQ) and Strychnobiflavone (SBF) | *Strychnos pseudoquina* | Bark of stem | Loganiaceae | HSV-1 (KOS strain) and HSV-2 (333 strain) | SEAE (standardized ethyl acetate extract) and SBF (strychnobiflavone) interfere with different HSV replication cycle specially adsorption, post adsorption and penetration and β and γ viral protein expression | *In vitro* | 108 |
|  | Ellagic acid, Ggallic acid | *Lagerstroemia speciosa* | Leaf and Stem | Lythraceae | HIV-1 | Protease and reverse transcriptase (Rtase) activity inhition | *In vitro* | 88 |
|  | Ellagitannin and Gallotannin | *Punica granatum* | Fruit peel |  | HSV-2 | Binds MTP (multiple target proteins) with lesser total free energy binding value and strong H2 bond interaction with selected targets | *In vitro* | 109 |
|  | Polyphenol | *Punica granatum* | Fruit juice |  | Influenza A virus | Fruit extract inhibits the virus proliferation in single-cycle growth conditions | *In vitro* | 110 |
|  | Honokiol (Lignan Biphenol) | *Magnolia officinalis* | Bark or Seed cones (fruit of conifers) | Magnoliaceae | Dengue virus Type 2 | Honokiol inhibits the intracellular DENV-2 replicon, suppresses the replication of DENV-2 in baby hamster kidney (BHK) and human hepatocarcinoma Huh7 cells | *In vitro* and *Clinical application* | 111 |
|  | Anthocyanin | *Althaea officinalis* | Leaf | Malvaceae | Influenza A virus H5N1 | NR | *In vitro* | 112 |
|  | Alkaloids, Flavonoids, Tannins, Volatile oils and Glycosides | *Cissampelos pareira Linn* | Aerial parts | Menispermaceae | Dengue virus types 1-4 (DENV-1-4) | Alcoholic extract revealed a potent inhibitor of all four DENVs (……) in cellbased assays, assessed in terms of viral NS1 antigen secretion using ELISA, as well as viral replication, based on plaque assays | *In vitro* and *In vivo* | 113 |
|  | Flavonoids, Tannins, Terpenes, Saponins and Nitrogenous compounds | *Artocarpus integrifolia* | Leaf, Flower, Seed and Bark | Moraceae | (SA-11) and human (HCR3) rotaviruses | inhibit the rotavirus propagation, protecting the cell cultures against virus invasion | *In vitro* | 33 |
|  | Flavonoids , Rutin, Kaempferol 3-O-rutinoside and Kaempferol 3-O-robinobioside | *Ficus benjamina* | Leaf |  | HSV-1 and HSV-2), Varicella Zoster Virus (VZV | NR | *In vitro* | 114 |
|  | N-argenine, Luteolin, Caffeic acid | *Ficus carica* | Fruit latex |  | HSV-1, HSV-1, ECV-11 and ADV, influenza virus | Viral adsorption and penetration in the cells were prevented and intracellular replication was inctivated | *In vitro* | 115 |
|  | Flavonoids, Tannins, Saponins, Alkaloids and Steroids/triterpenoids | *Ficus religiosa* | Bark of stem |  | HSV-2 | Chloroform extract targets cell-surface and intracellular components acted on HSV-2 replicative cycle | *In vitro* | 116 |
|  | NR (Other articles contain phytochemical report) You can include the phytochemicals name. because they are composed of different compunds and we want to mention the compound name :) | *Ficus septica* | Leaves, Stem, Fruit, Heartwood |  | Dengue virus types 1-4 (DENV-1-4) | NR | *In vitro* | 117 |
|  | Tannins, Flavonoid, Saponin, Glycoside | *Ficus sycomorus* | Stem, Bark and Leaf | Moraceae | HSV-1 | NR | *In vitro* | 56, 118 |
|  | Alkaloids, Tannins, Phenolics, and Saponins | *Moringa peregrina* | Seed | Moringaceae | HSV | NR | *In vitro* | 47 |
|  | Flavonoids | *Myristica fragrans* | Seed | Myristicaceae | Human (HCR3) rotaviruses | Virus propagation and virus invasion inhibited in cell cultures | *In vitro* | 33 |
|  | Tannins and Flavonoids | *Psidium guajava* | Leaf | Myrtaceae | Simian (SA-11) virus | NR | *In vitro* | 33 |
|  | Sesquiterpenes, Monoterpenes, Hydrocarbon, and Phenolic compounds, Eugenyl acetate, Eugenol, and β-caryophyllene | *Syzygium aromaticum L.* | Flower bud |  | HSV and HCV | By preventing viral replication and reducing the viral infections | *In vitro* | 119 |
|  | Harmine | *Peganum harmala* | Seed | Nitrariaceae | HSV-2 | Virus replication was inhibited by virucidal effect during and after virus penetration | *In vitro* | 120 |
|  | Paeoniflorin, Monoterpene Glycosides, Albiflorin, Benzoylpaeoniflorin, Gallic acid, Ethyl gallate | *Paeonia delavayi* | Root | Paeoniaceae | Influenza virus | Through strong anti -neuraminidase (NA) activity | *In vitro* | 121 |
|  | Oxypaeoniflorin, Albiflorin, Paeoniflorin, Benzoic acid, and Paeonol | *Paeonia lactiflora* | Root |  | Influenza virus A/WSN/33 (H1N1) | Viral hemagglutination inhibititon and blockage of viral binding and penetration into host cells | *In vitro* and *In vivo* | 122 |
|  | Flavonoids, Tomentin A, B C, D, and E | *Paulownia tomentosa* | Fruit | Paulowniaceae | SARS-CoV papain-like protease (PLpro) | Papain-like protease (PLpro) inhibition | *In vitro* | 123 |
|  | Highly oxygenated norbisabolane sesquiterpenoids, phyllanthacidoid acid, Methyl ester | *Phyllanthus acidus* | Bark and Root | Phyllanthaceae | Hepatitis B Virus | Ellagic acid, a phenolic compound inhibits HBeAg secretion | *In vitro* | 124 |
|  | Alkaloids, Flavonoids, Lignans, Phenols and Terpenes | *Phyllanthus amarus* | Root and Leaf |  | HCV | Reduced HCV RNA level by HCV protein expression inhibition | *In vitro* | 125 |
|  | Geraniin, Rutin, Gallic acid, Caffeolquinic acid, Corilagen, Galloylglucopyronoside, Digalloylglucopyronoside and Quercetin glucoside | *Phyllanthus amarus* | Harvested Plant |  | Acyclovir-resistant HSV strains, hepatitis B virus (HBV), HCV and HIV | NR | *In vitro* | 126 |
|  | Geraniin, Rutin, Gallic acid, Caffeolquinic acid, Corilagen, Galloylglucopyronoside, Digalloylglucopyronoside and Quercetin glucoside | *Phyllanthus niruri* | Harvested plant | Phyllanthaceae | Acyclovir-resistant HSV strains, hepatitis B virus (HBV), HCV, HIV | Virus eproduction inhition | *In vitro* | 126 |
|  | Trigalloylglucopyronoside, Quercetin rhamnoside, Geraniin, Rutin, Gallic acid, Caffeolquinic acid, Corilagen, Galloylglucopyronoside, Digalloylglucopyronoside and Quercetin glucoside | *Phyllanthus urinaria* | Harvested plants |  | Acyclovir-resistant HSV strains, hepatitis B virus (HBV), HCV and HIV | serotype specific inhibition against HSV-1 and HSV-2 | *In vitro* | 126 |
|  | Quercetin rhamnoside, Geraniin, Rutin, Gallic acid, Caffeolquinic acid, Corilagen, Galloylglucopyronoside, Digalloylglucopyronoside and Quercetin glucoside | *Phyllanthus watsonii* | Harvested plant |  | Acyclovir-resistant HSV strains, hepatitis B virus (HBV), HCV and HIV | NR | *In vitro* | 126 |
|  | lignin-carbohydrate complexes (LCC) | *Pinus yunnanensis* | Cone (contains the reproductive organs | Pinaceae | HIV-1 | Fusion inhibition of normal cells and HIV-1 infected cells, and recombinant HIV-1 reverse transcriptase activity inhibition | *In vitro* | 127 |
|  | Flavonols, Flavonol glycosides, Flavonol glycoside gallates, Flavones, Flavanones, and Flavan-3-ols | *Limonium sinense* | Underground part/Root | Plumbaginaceae | HCV | Blocking the early viral entry | *In vitro* | 128 |
|  | Plumbagin, Allicin, Carbohydrates, Flavonoids, Proteins, Saponins, Fats and oils, Alkaloids, Steroids, Phenols, and Tannins | *Plumbago indica* | Root |  | Influenza A (H1N1) | Blocks the viral adsorption to cells | *In vitro* and *In silico* | 129 |
|  | Emodin (3-methyl-1,6,8-trihydroxyanthraquinone) - an anthraquinone derivative | *Rheum tanguticum* | Root | Polygonaceae | HSV-1 and HSV -2 | Emodin may inhibit HSV biological synthesis rather than directly inactivating the viruses/ blocking their absorption to the susceptible cells | *In vitro* and *In vivo* | 130 |
|  | sulfated polysaccharide fraction | *Ardisia chinensis Benth* | Whole plant | Primulaceae | Coxsackie B3 Virus | NR | *In vitro* | 131 |
|  | Benzoquinones and embelin | *Embelia ribes* | Fruit | Primulaceae | Influenza virus A/Puerto Rico/8/34 (H1N1) | Eembelin (principle constituent) binding site is located at viral hemagglutinin receptor-binding domain which suppressed the virus replication at early stages of the viral life cycle and showed inhibitory activity to extracellular virions. | *In vitro* and *In silico* | 132 |
|  | Jatrorrhizine, Palmatine and Berberine | *Coptis chinensis* | Root | Ranunculaceae | HCV | Inhibits Early Viral Entry, blocks HCV viral attachment and entry or fusion into the host cells | *In vitro* and *In vivo* | 133 |
|  | Jatrorrhizine, Palmatine and Berberine | *Coptis deltoidea* | Root |  | HCV | Inhibits the Early Viral Entry Steps, blocks HCV viral attachment and entry/fusion into the host cells | *In vitro* and *In vivo* | 134 |
|  | Flavonoids (catechin, Hyperoside, Quercitrin, quercetin and rutin), Tannins and Triterpenoids | *Agrimonia pilosa* | Whole plant (Dried) | Rosaceae | Influenza viruses (H1N1 and H3N2) | flavonoids such as catechins, quercetin, rutin, quercitrin and hyperoside are responsible for inhibition of the reverse transcriptase activity of HIV and are active against RNase activity and recombinant HIV-1 | *In vitro* and *In vivo* | 135 |
|  | Hydroxycinnamic acids, Eriodictyol, Isorhamnetin, Quercetin, Kaempferol, Isorhamnetin,, Epicatechin, Catechin | *Prunus dulcis* | Peel of seed |  | HSV-1 | HSV-1 replication hampered by blocking virus binding to the cell | *In vitro* | 136 |
|  | Chlorophyll catabolites, Pheophorbide A and Pyropheophorbide A | *Morinda citrifolia* | Leaf | Rubiaceae | HCV | HCV infection inhibited at post-entry step by the action Pheophorbide A and pyropheophorbide A | *In vitro* | 137 |
|  | Saponins, Flavonoids and Alkaloids, | *Pavetta tomentosa* | Leaf |  | Dengue virus (DENV) | NR | *In vitro* and *in vivo* | 138 |
|  | Saponins, Favonoids and Alkaloids, | *Tarenna asiatica* | Leaf |  | Dengue virus (DENV) | NR | *In vitro and In vivo* | 138 |
|  | Carbohydrates, Glycosides, Amino acids, Proteins, Tannins, Flavanoids, and Ahytosterols | *Aegle marmelos* | Fruit (unripen) | Rutaceae | Human coxsackieviruses B1-B6, Rotavirus SA-11 | NR | *In vitro* | 139 |
|  | Triterpenes, Tannins, Flavonoids and Carbohydrates | *Dimocarpus longan* | Leaf | Sapindaceae | HCV (genotype 2a strain JFH1) | Anti-HCV activity through a direct virucidal effect and by reduction of HCV infectivity at the post-entry step is due the direct inactivation of virion released from infected cells | *In vitro* | 140 |
|  | Manassantin B | *Saururus chinensis Baill* | Aerial part | Saururaceae | Coxsackievirus B3 (CVB3) | By activation of STING/TBK-1/IRF3 signalling pathway | *In vitro* | 141 |
|  | Organic acids, Terpenoids and Flavonoids | *Illicium verum Hook. f.* | Fruit | Schisandraceae | Grouper iridovirus infection (GIV) | NR | *In vitro* and *In vivo* | 142 |
|  | Nilocitin, Ellagic acid, Gallic acid, Flavonoids | *Tamarix nilotica* | Aerial part | Tamaricaceae | HSV | NR | *In vitro* | 47, 143 |
|  | Diterpenoids, Biflavonoids (Biflavone amentoflavone, Apigenin, Luteolin and Quercetin) | *Torreya nucifera* | Leaf | Taxaceae | SARS-CoV 3CLpro | Extract inhibited the virus and molecular docking supports the inferences drawn from the enzymatic assay, revealing inhibitory action of biflavones on the virus | *In vitro* and *In silico* | 144 |
|  | Epigallocathechin-3-gallate, polyphenol catechins such as C, EGC, ECG and EC. | *Camellia sinensis* | Natural green tea extract | Theaceae | HIV, HTLV-1, HCV, Influenza and HBV | Expression level of HBV DNA, HBsAg and HBeAg reduction | *In vitro* and clinical applicat-ion | 145, 146 |
|  | Friedelolactones, 2β-hydroxy-3, 4-seco-friedelolactone-27-oic acid Flavonoids, Coumarins , Terpenoids, Sterols, Polypeptides | *Viola diffusa* | Whole plant | Violaceae | Hepatitis B Virus | NR | *In vitro* | 147 |
|  | Diarylheptanoids, monoterpenes, Sesquiterpenoid, Flavonoids, and Chalcones | *Alpinia katsumadai* | Seed | Zingiberaceae | influenza virus type A | Blockage of viral binding to the cell receptor and viral replication inhibition | *In vitro* | 148 |
|  | Allyl disulfide, allyl trisulfide, allyl (E)-1-propenyl disulfide, allyl methyl trisulfide and diallyl tetrasulfide | *Allium sativum L.* |  | ‎Amaryllidaceae | SARS-CoV‑2 | inhibition of the ACE2 and PDB6LU7 proteins | Molecular docking | 149 |
|  | Torilin | *Torilis fructus* | Plant Material | Apiaceae | SARS-CoV | Reduce intracellular viral RNA levels with comparable reductions in viral proteins and MHV-A59 production | in vitro | 150 |
|  | Lycorine | *Lycoris radiata* | Stem | Amaryllis | SARS-CoV | inhibition effects on virus-induced CPE | in vitro | 151 |
|  | Acanthoside, Chiisanoside and phytosterol | *Acanthopanacis cortex* | Plant Material | Araliaceae | SARS-CoV | Reduce intracellular viral RNA levels with comparable reductions in viral proteins and MHV-A59 production | in vitro | 150 |
|  | carvacrol and a-pinene | *Anthemis hyaline* | flowers and buds | Asreraceae | SARS-CoV | Decrease replication through the involvement of TRP genes family | in vitro | 152 |
|  | Quinone-methide triterpenes (celastrol, pristimerin, tingenone and iguesterin) | *Tripterygium regelii* | stem root | Celastraceae | SARS-CoV | SARS-CoV 3CLpro inhibitors | in vitro | 153 |
|  | a-pinene and b-myrcene | *Juniperus oxycedrus* | Berry | Cupressaceae | HSV-1 | - | in vitro | 154 |
|  | Brazilein, Brazilin | *Caesalpinia sappan* | - | Fabaceae | SARS-CoV-2 | Highe binding affinities with SARS-CoV-2 protease (PDB:6LU7), Spike glycoprotein-RBD (PDB:6LXT), and PD-ACE2 (PDB:6VW1) | Molecular docking | 155 |
|  | Anthraquinones including emodin, physcion and rhein | *Cassia tora* | seed | Fabaceae | SARS-CoV | inhibits the 3CL protease activity of SARS-CoV | in vitro | 156 |
|  | Flavonoids (bavachinin, neobavaisoflavone, isobavachalcone, 40-O-methylbavachalcone, psoralidin and corylifol A) | *Psoralea corylifolia* | seed | Fabaceae | SARS-CoV | papain-like protease inhibition | in vitro | 150 |
|  | triterpenoids | *Gentiana scabra* | Rhizome | Gentianaceae | SARS-CoV | inhibits the 3CL protease activity of SARS-CoV | in vitro | 156 |
|  | procyanidin A2 and procyanidin B1 | *Cinnamomum verum* | inner bark | Lauraceae | SARS-CoV | inhibits the internalization of TfR indicating the interference of clathrin-dependent endocytosis | in vitro | 157 |
|  | b-ocimene, 1,8-cineole, a-pinene, and b-pinene | *Laurus nobilis* | Berry | Lauraceae | SARS-CoV | - | in vitro | 154 |
|  | luteolin and quercetin | *Taxillus chinensis* | Stem | Loranthaceae | SARS-CoV | inhibits the 3CL protease activity of SARS-CoV | in vitro | 156 |
|  | Emodin (anthraquinone ) | *Polygonum multiflorum* | Root tuber, Vines | Polygonaceae | SARS-CoV | blocked the S protein and ACE2 interaction | in vitro | 158 |
|  | Emodin (anthraquinone ) | *Rheum officinale* | Root tuber | Polygonaceae | SARS-CoV | blocked the S protein and ACE2 interaction | in vitro | 158 |
|  | anthraquinones | *Rheum palmatum* | roots and rhizomes | Polygonaceae | SARS-CoV | high level of anti-SARS-CoV 3CL protease activity | in vitro | 159 |
|  | thymoquinone, qsimen, karvakrol, t-anetol, 4-terpineol and longifoline | *Nigella sativa* | seeds | Ranunculaceae | SARS-CoV | Decrease replication through the involvement of TRP genes family | in vitro | 152 |
|  | flavonoids, limonene and linalool | *Citrus sinensis* | peels | Rutaceae | SARS-CoV | Decrease replication through the involvement of TRP genes family | in vitro | 152 |
|  | Nafamostat, Lopinavir | *Alpinia galanga* | - | Zingiberaceae | SARS-CoV-2 | Highe binding affinities with SARS-CoV-2 protease (PDB:6LU7), Spike glycoprotein-RBD (PDB:6LXT), and PD-ACE2 (PDB:6VW1) | Molecular docking | 155 |

NR: Not reported
